# Discovery of interconnected causal drivers of COVID-19 vaccination intentions in the US using a causal Bayesian network

**DOI:** 10.1038/s41598-023-33745-4

**Published:** 2023-05-16

**Authors:** Henry Fung, Sema K. Sgaier, Vincent S. Huang

**Affiliations:** 1 Surgo Health, Washington, DC USA; 2 Surgo Ventures, Washington, DC USA; 3 Department of Global Health, University of Washington, Seattle, WA USA

**Keywords:** Public health, Health policy

## Abstract

Holistic interventions to overcome COVID-19 vaccine hesitancy require a system-level understanding of the interconnected causes and mechanisms that give rise to it. However, conventional correlative analyses do not easily provide such nuanced insights. We used an unsupervised, hypothesis-free causal discovery algorithm to learn the interconnected causal pathways to vaccine intention as a causal Bayesian network (BN), using data from a COVID-19 vaccine hesitancy survey in the US in early 2021. We identified social responsibility, vaccine safety and anticipated regret as prime candidates for interventions and revealed a complex network of variables that mediate their influences. Social responsibility’s causal effect greatly exceeded that of other variables. The BN revealed that the causal impact of political affiliations was weak compared with more direct causal factors. This approach provides clearer targets for intervention than regression, suggesting it can be an effective way to explore multiple causal pathways of complex behavioural problems to inform interventions.

## Introduction

Two years after COVID-19 was declared a global pandemic, it remains the greatest public health crisis facing the United States. By December 2021, the country had suffered 48 million cases and 770,000 deaths^1^. The development and availability of vaccines has been a crucial step in preventing the worst clinical effects of the coronavirus. However, the pace of vaccination has stagnated, falling from a peak of 3 million new doses per day in April 2021 to 1.5 million daily in December 2021^2^. Despite efforts to boost vaccination rates, a large proportion (29.6%) of the eligible US population remain unvaccinated^1^—many by choice—and thus vulnerable to severe illness and death should they become infected.

COVID-19 vaccine hesitancy is a complex behavioural problem^3^. There is no shortage of hypotheses on why: vaccine safety and side-effect concerns, politics, disinformation, race, perceptions, emotions, social norms, individual influences, knowledge, and economic factors have all been proposed^4–14^. Since randomised control trials (RCT) are challenging or impossible, many quantitative studies have sought to identify the determinants of vaccine hesitancy from observational data using descriptive statistics and linear regression models^7–11^. These studies suggest that perceived COVID-19 risk, conservative-leaning political views, prior vaccine usage and attitudes, and vaccine safety concerns are strong predictors of vaccine hesitancy^7–11^. A substantial body of qualitative research has focused on using qualitative data from in-depth interviews, social media conversations and content, or open text response in surveys to understand and capture the points of view of general populations^15–18^ and subpopulations in the US^19–21^ (e.g., Black and Latino Americans) who remained unvaccinated without predetermining those points of view through prior selection of survey topics. These studies suggest that concerns about potential vaccine side-effects, mistrust of the healthcare system and pharmaceutical companies, financial issues, and myths and misconceptions about COVID-19 affect the intent to get vaccinated. This current study was built on these deep explorations of vaccine hesitancy context as an attempt to abstract and structuralize the hierarchy and interactive nature of these factors in the US.

These studies, however, have some limitations. First, many quantitative studies focus more on sociodemographic differences than on underlying beliefs and barriers^4–6^. Second, correlation does not imply causation^22, 23^. For instance, with observational data, the potential to misattribute correlation to causation due to confounding (i.e., when a spurious association between two variables is found due to a third variable having an influence on both of them) is often acknowledged but is challenging to address^22, 24, 25^. Although statistical corrections for these effects are possible, the experts with the required knowledge may suffer from subjective bias or be prejudiced by hypotheses^25–27^. Third, many studies focus more on identifying predictors of the outcome of interest^7, 9, 14, 28^ and less on understanding how these predictors interact at a systems level to give rise to the outcome (causal inference). There are likely multiple intersecting paths and intermediary steps by which causal factors can influence behaviours. Understanding this could be critical to inform more nuanced and precise interventions. However, statistical causal inference techniques (e.g., propensity score matching, regression discontinuity design) are not designed to test multiple, connected causal hypotheses simultaneously^24^ (e.g., A causes B, B causes C, but A also directly causes C etc.). As a result, it is difficult to explore multiple causal pathways of behavioural outcomes with these approaches. An easier conceptualisation of the multi-causal complexities of human behaviours is a structural causal model (SCM) such as a structural equation model (SEM)^8, 28^. However, SEM requires specifying what variables interact and how they do so, which means that the causal structure is completely specified a priori, not by data.

**Figure 1:**
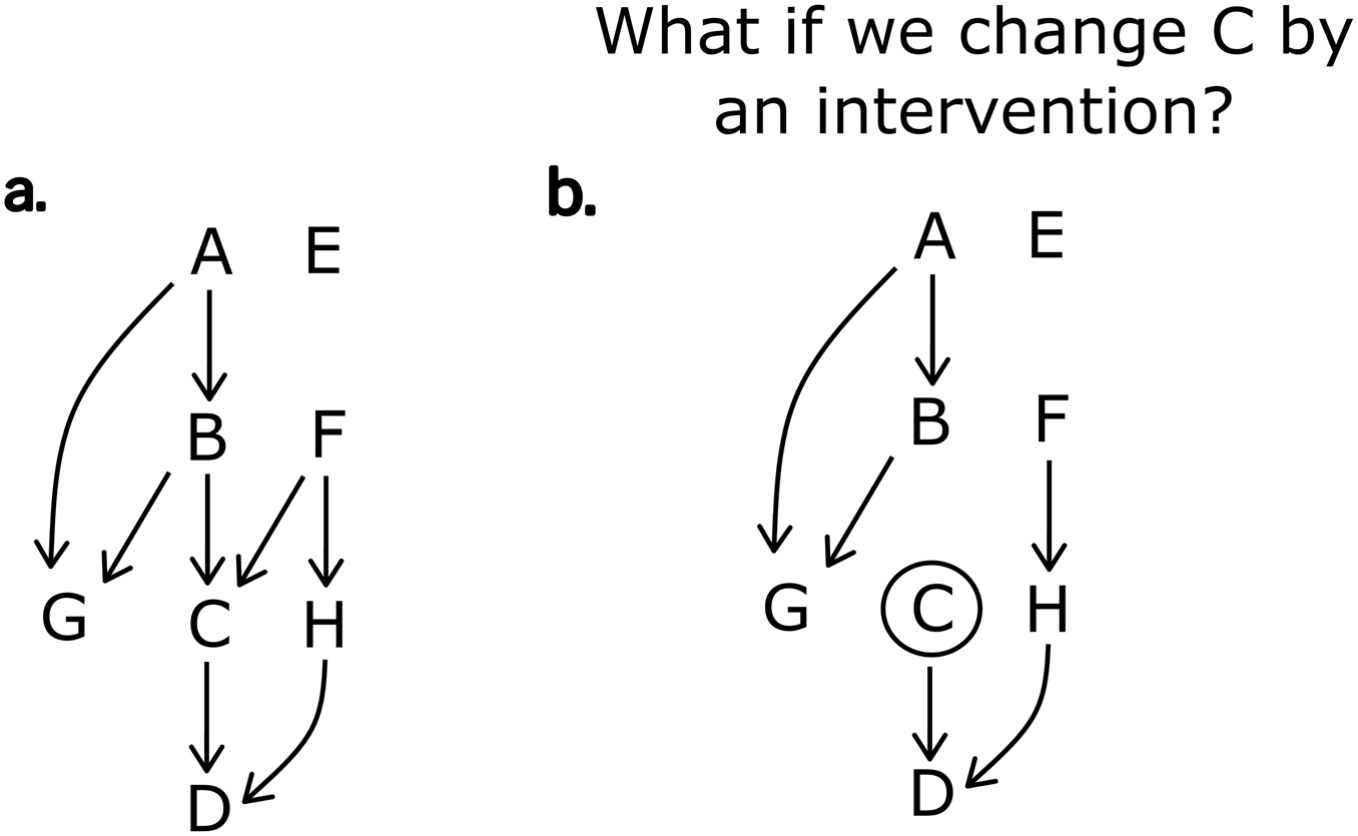
An example of a simple directed acyclic graph (DAG) and an interventional query. (**a**) Variables are depicted as a set of nodes, and the probabilistic conditional dependency among variables is depicted by a set of directed arrows. Here, C is dependent on A, B and F, but not on E, D, G or H. B and F are direct causes of C, while A is an indirect cause of C via B. E is not causally related to any other variables. F can impact D via C or H. F is a common cause to both C and D; thus, C’s effect on D is confounded by F. B can be seen as an instrumental variable for the effect of C on D^32^. (**b**) Depiction of an interventional query on “C.” This is the equivalent of removing any connections upstream of C, and then estimating the probabilities of different outcomes of another variable due to a change in C.

Recent advances in machine learning have spawned a class of algorithms called causal discovery to autonomously construct a type of SCM called causal Bayesian Network (BN) which can be combined with human expert insights or findings from qualitative research as needed. Graphically, a BN is depicted by a Directed Acyclic Graph (DAG)^29, 30^ (see Fig. 1a for an example). Based on the statistical conditional dependencies amongst variables, BN lends itself to causal inference to estimate how much any variable would change if the state or value of another variable is changed as a result of an intervention (this is known as “what-if analyses” or “interventional queries”) (Online Appendix A)^31^. To distinguish this from typical correlation analysis or predictive models (i.e., observational queries), imagine trying to attribute an increase in vaccination rate (say, “D” in Fig. 1) to 100 extra vaccination sites (“C”). In a predictive model, the observed number of vaccination sites could be the result of a broader intervention such as increased funding (“F”) that also affects other potential variables such as community outreach (“H”). Therefore, any increase in vaccination rate cannot be attributed solely to the number of vaccination sites.

By contrast, in an interventional query we forcibly replace the value of one variable to see what change it would bring to the outcome of interest. In this case, the specific number of extra vaccination sites no longer depends on any other variables like funding. Graphically, this is equivalent to removing any connections upstream of “C” (i.e., graph surgery) and then estimating the likely outcome of the outcome variable of interest due to a change in “C” (Fig. 1b). Formally this is known as do-calculus first proposed by Judea Pearl^31^. Thus, conceptually, interventional queries are like comparing treatment groups with control groups in a virtual RCT, whereas observational queries are more akin to using correlations to predict expected observation (without accounting for all confounding biases). This makes interventional queries far more informative for the purpose of identifying targets of intervention design. Notably, unlike an experiment, where a new RCT is needed to test each new causal hypothesis, the same model can be used to explore “causes of cause” if the immediate cause is not directly actionable, or if one would like to investigate upstream or alternative causal paths.

We conducted the Surgo COVID-19 Vaccine Survey (Online Appendix B) in early 2021, when vaccines were first made widely available in the US, collecting a wide range of psycho-behavioural data on variables that may drive COVID-19 vaccine intention. With this data and BN, we aimed to gain systems-level insights into the complex causal pathways influencing vaccine intent, without a priori model specification. Here we aim to (1) identify the complexities and the mechanisms leading to COVID-19 vaccine intention (i.e., “When a vaccine for COVID-19 is available to you, how likely are you to take it?”), and (2) contrast this with the conclusions that would be reached via logistic regression from the same data. Finally, we suggest several strategies to increase vaccine intent.

## Results

### The causal pathways are complex for vaccine intention, with beliefs in social responsibility, vaccine safety and anticipated regret forming the most direct causes

**Figure 2:**
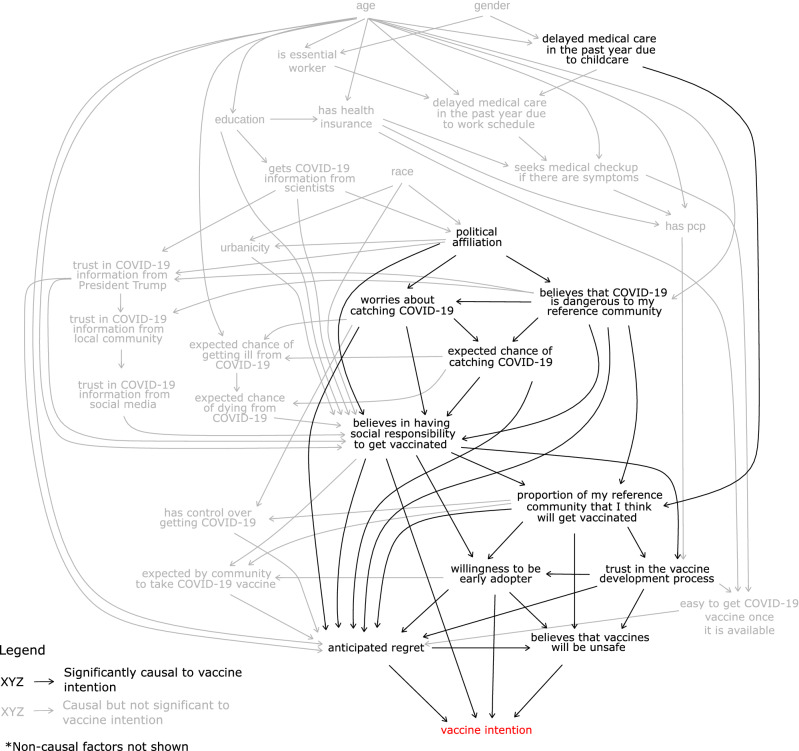
The BN structure learnt (developed) from the Surgo COVID-19 Vaccine Survey data, depicted as directed acyclic graph (DAG). For clarity, we only include nodes that are direct (immediately upstream) or indirect causes (further upstream) of vaccine intention. Significant causes are those whose change would significantly alter vaccine intention (p-value < 5%) when we perform simulations. The direct causes of vaccine intention are mostly beliefs and perceptions, and social norms. Direct causes are in turn driven by remote causes, which consist mostly of structural factors, influencers and outcome expectations.

The resulting BN of 45 categorical variables from the Surgo COVID-19 Vaccine Survey reveals a rich network of inter-dependencies between the causal factors of vaccine intention: demographics, structural factors, social influences, beliefs and perceptions, emotions, influencers and behaviours (Fig. 2 and Table 1). For convenience, we call any variables that are upstream of vaccine intention in a directed path *causal factors* to vaccine intention. Those that have statistically significant estimated causal effects (p-value < 5%) on vaccine intention are called *significant causal factors* of vaccine intention. For clarity, we omit the non-causal factors (Online Appendix C, Table C3) in Fig. 2 but the full DAG can be found in Online Appendix D.

**Table 1:** Variables included in the Bayesian Network, along with their scales and the causal factor category that they belong to.

Variable name	Scale
Outcome expectation	
Expected chance of getting seriously ill from COVID-19	0: Almost zero or low; 1: moderate; 2: high or almost certain
Expected chance of getting COVID-19 with no vaccine	0: Almost zero or low; 1: moderate; 2: high or almost certain
Expected chance of dying from COVID-19 with no vaccine	0: Almost zero or low; 1: moderate; 2: high or almost certain
Expected chance of getting long-term side-effects from COVID-19 vaccine	0: Almost zero or low; 1: moderate; 2: high or almost certain
Emotions	
Worries about catching COVID-19	0: Not at all or not much; 1: moderate; 2: a great deal
Regret if I did not take COVID-19 vaccine (i.e. anticipated regret)	0: No; 1: yes
Beliefs and perceptions	
Believes COVID-19 vaccine is free	0: No; 1: yes
Believes COVID-19 vaccine is tested for safety and effectiveness in people of my race	0: Disagree; 1: agree; 2: neither agree or disagree
Believes people of my race are fairly treated in a health-care setting	0: No; 1: yes
Trust in the vaccine development process of pharmaceutical firms (i.e., trust in the vaccine development process)	0: Disagree; 1: agree
Believes COVID-19 vaccine will be unsafe	0: No; 1: yes
Believes COVID-19 testing is too rushed	0: No; 1: yes
Believes in having responsibility to get vaccinated for COVID-19 to protect others (i.e., social responsibility)	0: No; 1: yes
Believes that a tracking chip is implanted in me through vaccine	0: No; 1: yes
Has control over getting COVID-19	0: No; 1: yes
COVID-19 is perceived to be dangerous to my community	0: No; 1: yes
Influencers/channels	
Believes in COVID-19 information from Trump	0: Disagree; 1: agree; 2: sometimes agree or disagree
Believes in COVID-19 info from public health organisations	0: Disagree; 1: agree; 2: sometimes agree or disagree
Believes in COVID-19 information from my physician	0: Disagree; 1: agree; 2: sometimes agree or disagree
Believes in COVID-19 information from my local community	0: Disagree; 1: agree; 2: sometimes agree or disagree
Believes in COVID-19 information from my social media contacts	0: Disagree; 1: agree; 2: sometimes agree or disagree
Gets information on COVID-19 from left-wing media	0: No; 1: yes
Gets information on COVID-19 from Fox News	0: No; 1: yes
Gets information on COVID-19 from social media	0: No; 1: yes
Gets information on COVID-19 from scientists	0: No; 1: yes
Social influence	
Proportion of community that I think will take the COVID-19 vaccine	0: None or fewer than half; 1: more than half or all
Reference community expects me to take COVID-19 vaccine	0: No; 1: yes; 2: not sure or no answer
Reference community considers COVID-19 a serious threat	0: No; 1: yes; 2: not sure
Structural enablers/barriers	
Has health insurance	0: No; 1: yes
Have primary care physician	0: No; 1: yes
Easy to get COVID-19 vaccine once it is available	0: No; 1: yes
Delayed medical care due to cost	0: No; 1: yes
Delayed medical care due to work schedule	0: No; 1: yes
Delayed medical care due to childcare	0: No; 1: yes
Demographics	
Gender	0: Man; 1: woman
Race	0: White; 1: black; 2: other minorities
Political affiliation	0: Republican; 1: democrat; 2: independent or other
Education	0: High school or less; 1: some college or associates; 2: bachelor's degree or more
Age	0: 18–34; 1: 35–64; 2:65 or over
Is essential worker	0: No; 1: yes
Urbanicity	0: Urban; 1: rural
Income	0: Less than $30,000; 1: $30,000 to under $100,000; 2: $100,000 or more
Behaviours	
Seek check-up if I have symptoms in general	0: No; 1: yes
Intentions	
Willing to take the COVID-19 vaccine in the first three months (i.e., early adopter)	0: No; 1: yes; 2: unsure
Dependent variable	
Vaccine intention: when a vaccine for COVID-19 is available to you, how likely are you to take it?	0: Low; 1: moderate; 2: high

When asked about their “reference community”, respondents mostly specified their immediate family and friends.

We found 30 variables that are either direct (immediately upstream) or indirect (further upstream) causes of vaccine intention. Remote causes (i.e., several nodes upstream of *vaccine intention*) tend to be influencers, structural factors and outcome expectations. These variables in turn drive the more direct causes of *vaccine intention*, which are dominated by beliefs and perceptions and by social influences (which include social norms and societal expectations). There are four direct causes of vaccine intention with significant causal effects: *the belief that vaccines will be unsafe; the belief in having social responsibility to get vaccinated for COVID-19 to protect others (social responsibility); feeling regret if one did not take the COVID-19 vaccine and then subsequently contracted COVID-19 (anticipated regret);* and *willingness to take the COVID-19 vaccine in the first three months of availability (early adopter)*. *Early adopter* is a very close proxy of vaccine intention. Variables found not to be causal of vaccine intention include *getting information about COVID-19 from the left-wing media or from Fox News; delayed in seeking medical care in the past year due to cost; income;* and the *belief that COVID-19 vaccine testing is rushed*.

Unlike regression analysis, we could identify from the DAG several sequences of cause-and-effect mechanisms (a.k.a. causal pathways) through which a given intervention ultimately affects vaccine intention. This suggests that there are multiple means for practitioners to influence vaccine intention directly or indirectly. *Social responsibility* and *anticipated regret* are particularly important; in addition to having direct effects on vaccine intention, these causes also mediate the effects of many demographic factors (e.g., *age*, *urbanicity* and *political affiliation*), as well as the effects of perceived risk of COVID-19 and influencers, on vaccine intention. In particular, *social responsibility* centrally affects several important factors downstream—*willingness to be an early adopter*, *anticipated regret* and *the belief that vaccines will be unsafe.* Thus, *social responsibility* should be considered a primary target for interventions.

Further upstream, we found that *believing that COVID-19 is dangerous to one’s community* is a remote but significant causal factor of vaccine intention that drives many factors downstream: *expected chance of catching COVID-19*, *the level of worry about catching COVID-19*, *the proportion of the community that I think will take the vaccine,* and importantly, *anticipated regret* and *social responsibility* mentioned above.

### Convincing people to have the social responsibility to get vaccinated has the greatest effect on vaccine intention

To estimate the effect size caused by a given intervention, we compute the interventional odds ratio (OR), which estimates the increase in odds of an outcome given a hypothetical and active change in an upstream variable from a reference level^31, 34^. Note that in performing the interventional query, we are not simulating an empirical intervention, but a hypothetical change of the value of the specific upstream variable (Online Appendix A).

**Figure 3:**
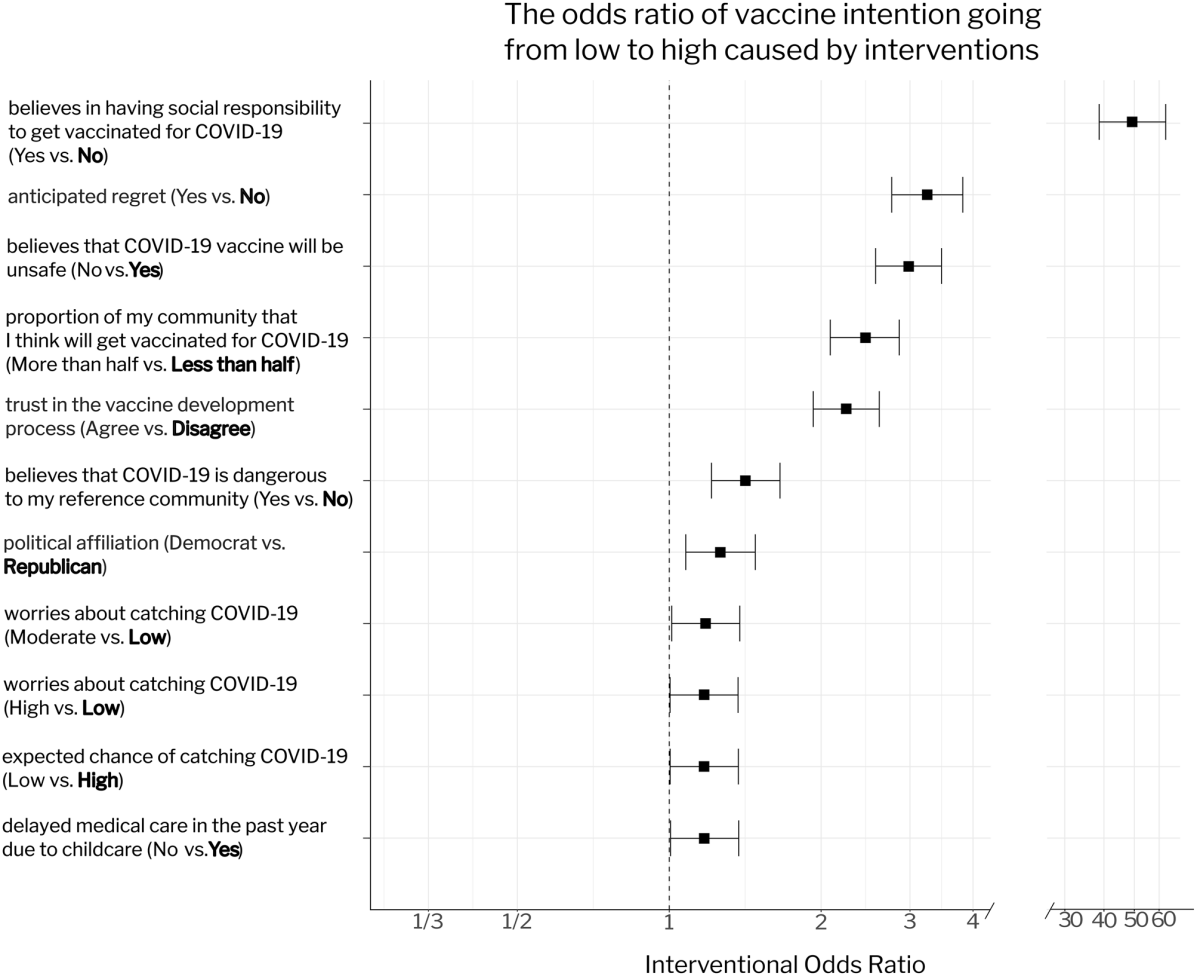
The interventional odds ratio of causal factors that have statistically significant estimated causal effects (at the 5% level) on vaccine intention. The bold text in the y-axis labels indicates the reference level of a given variable, and the error bars are the 95% confidence interval of the odds ratio estimate. The results suggest that making people believe that it is their social responsibility to get vaccinated to protect others, triggering anticipated regret for not taking the vaccine, making people believe that the majority of their community are taking the vaccine, and discouraging the belief that vaccines will be unsafe have the largest effect in increasing vaccine intention from low to high.

Figure 3 shows the interventional ORs and 95% confidence intervals (with numerical details in Online Appendix D, Tables D1–D7). Convincing people of the social responsibility to get vaccinated (from low to high social responsibility) has by far the largest effect on moving people from low to high levels of vaccine intention (OR = 49.33, 95% CI 38.64–62.99), followed by showing people that they may regret not taking the vaccine (OR = 3.24, CI 2.75–3.81). The effect sizes of these drivers suggest that messages emphasising *social responsibility and anticipated regret* could be leveraged even more than is currently happening, to change the minds of those with low vaccine intent.

Our analysis also suggests that encouraging people to believe that COVID-19 vaccines are safe (OR = 2.94, CI 2.56–3.45), promoting trust in the vaccine development process (OR = 2.24, CI 1.93–2.60), persuading people that COVID-19 poses a danger to members of their community (OR = 1.42, CI 1.216–1.66) and that the majority of their community will take the vaccine (OR = 2.44, CI 2.08–2.85) will motivate people with low vaccination intention. Similar causal factors were identified, but with a smaller interventional OR, for driving people with moderate vaccination intention to high vaccine intention (Fig. D2, and Tables D8–D14 in Online Appendix D).

### The causal factors of social responsibility, the belief that vaccines will be unsafe, and anticipated regret

Variables such as *social responsibility*, *the belief that vaccines will be unsafe* and *anticipated regret* may be seen as vague targets for interventions. We therefore leveraged a particular feature of BNs—that causes of one variable can be outcomes of another—and examined the “causes of cause” of vaccine intention without having to rebuild a new model (“Causes of cause” section, Tables E1–E16 in Online Appendix E).

We found that *social responsibility* and *anticipated regret* are driven by a set of factors related to the risk perception of COVID-19 to self and others (e.g., *expected chance of getting COVID-19 without vaccines, worry about getting COVID, belief that COVID is dangerous to one's community*). Additionally, *anticipated regret* is driven by a set of drivers related to social considerations and vaccine safety concerns. The individually small effects of these variables suggest that *social responsibility* and *anticipated regret* will require a more concerted effort to nudge (i.e., a simultaneous intervention on their multiple causes) as a means to increase vaccine intention.

The *belief that vaccines will be unsafe* is driven by *trust in the vaccine development process*, *proportion of my reference community that I think will get vaccinated* and *anticipated regret*. This suggests that practitioners can appeal to a mixture of facts (e.g., the clinical trials that vaccines undergo in their development) and emotions (e.g., anticipated regret) in addressing vaccine safety concerns.

### Using the same model to study the impact of political affiliation

There is a popular belief, in part propagated by the media, that Republicans are to be blamed for the low COVID-19 vaccine uptake in the US^4^. It is not without empirical support. The associations between vaccine hesitancy and political affiliation shown by Khubchandani et al., Viswanath et al.^35, 36^ and others^10, 37^, along with our own correlative analysis (Online Appendix F) have suggested that Republicans on average have higher vaccine hesitancy than Democrats. However, our causal analysis paints a more nuanced picture of the influence of political affiliation (Online Appendix G). Although social responsibility is an important and direct cause of vaccine intention, it is only weakly influenced by political affiliation (OR = 1.18, 95% CI 1.06–1.32). Instead, the effect of political affiliation on vaccine intention is mostly mediated by *worries about catching COVID-19* and *belief that COVID-19 is dangerous to my community*—both remote and weak causes of vaccine intention. For this reason, the estimated causal relationship between political affiliation and vaccine intention is small, and the strong observed association between these variables is likely due to confounders. Upon a closer look, *race* and *getting COVID-19 information from scientists*—a proxy for general trust in science—are significant causal factors of political affiliation (Online Appendix E, Table E16). Although individually neither of the factors are significantly causal to *both* political affiliation and vaccine intention, they could still confound the relationship between political affiliation and vaccine intention if they were to change simultaneously. Overall, the confounders would lead to an overestimation of the effect of political affiliation in non-causal analyses.

### Comparing the findings from BN and a multinomial logistic regression model

With the understanding that correlations alone do not imply causation, we contrast the findings from BN and a multinomial logistic regression model to highlight the recommendations that we might have reached if the correlative approach was used on the same dataset (Online Appendix C, Tables C1–C3). From the multinomial logistic regression model, 22 correlates are significantly associated with vaccine intention. By contrast, only 11 causal drivers from the BN have significant causal effects. This suggests that some of the significant associations estimated by regression would not be effective targets for interventions, as they are not supported by the causal analysis. There are several discrepancies between the results from the BN model and the regression model. First, while the regression model identifies beliefs in conspiracy theories, long-term side-effects from vaccines, and various sources of COVID-19 information to be determinants of vaccine intention, the BN identifies these variables either as non-causes or as remote and insignificant causal factors. Second, the belief in various information sources was found to be significantly associated with vaccine intention by the regression model but were not significant causal factors by the BN. Third, the regression model and BN differ in the type of vaccine safety concern that they identify as influential for vaccine intention. The regression model identifies *the belief that COVID-19 vaccine is tested for the safety of my race* and *the belief that COVID-19 testing is too rushed* as important concerns, while the BN suggests that *trust in the vaccine development process* is an important concern. The differences might be explained by several methodological differences between regression models and BN, as we describe in the Discussion.

## Discussion

Using a rich observational dataset from a nationally representative survey, we identified causal factors of COVID-19 vaccine intent in the United States and their mechanisms using machine learning and BN. We showed how insights generated from this approach can be much more nuanced and informative for intervention design than logistic regression alone.

The discrepancies between the BN and logistic regression models can be explained by the differences in complexity and modelling method (unsupervised structural learning in BN versus modeller-specified formulae). The BN models causal factors and vaccine intention as a complex system of causal interdependencies; thus, it can estimate the indirect or direct causal effects of these factors upon vaccine intention. In contrast, regression models often ignore the interdependencies among covariates and (falsely) treat each behavioural predictor as being directly and linearly associated with vaccine intention. However, numerous studies have found that interactions are possible among behavioural and demographic variables^38, 39^. There is also no evidence supporting the linearity of the associations between these predictors and vaccine intention. Since additivity and linearity—two important assumptions of linear regression—are likely violated, in addition to the fact that regression models only estimate associations rather than causation, it is unsurprising to see disparities between the findings of these two methods.

Our study suggests that practitioners could focus on designing interventions that promote social responsibility and anticipated regret, and dispel vaccine safety concerns. These factors have a much larger causal impact on vaccine intent than demographic attributes. Ideally, individual interventions should not be done in isolation, as shown by the interdependencies of various causal factors captured by the BN model.

While there is evidence of success from governments that have stressed the importance of getting vaccinated as a social responsibility and civic duty^40, 41^, and several studies have shown the positive association between social responsibility and vaccine intent and uptake^42–44^, the 49-fold magnitude of the impact of social responsibility relative to other strategies such as addressing vaccine safety concerns^45^ and leveraging descriptive social norms^46, 47^ is notable. Several factors in turn have significant effects on social responsibility: the expected chance of getting COVID-19 without vaccines, the level of worry about catching COVID-19, and the belief that COVID-19 is dangerous to one’s community. Since the individual effect of each of these drivers on social responsibility is small, a cocktail of interventions that targets them simultaneously may be necessary to promote it.

Less was known about the extent to which anticipated regret affects COVID-19 vaccination decisions. Merely asking whether someone would anticipate regret for not engaging in a health behaviour (e.g., skipping an annual physical exam) has previously been shown to be sufficient in motivating that behaviour^48^. A meta-analysis^49^ of 81 studies have shown that anticipated regret is positively associated with various health behaviours that ranges from cancer screening and physical activity to vaccination, Our results show that the causal effect of triggering anticipated regret is greater than that of addressing vaccine safety concerns. Notably, anticipated regret is driven by perceived risk of COVID-19 to self and others, social responsibility, norms and expectations, and trust in vaccine development. Collectively, these results suggest that to convince people that they will feel regret if they do not take the COVID-19 vaccine, they must first be convinced that the negative health or social consequences could be realised—but this must be done in a persuasive way that considers people’s needs for autonomy and self-determination, to avoid provoking resistance^50^.

Our findings support existing interventions that focus on addressing vaccine safety concerns^45^, such as communicating results of COVID-19 vaccine safety surveillance^51^ and low incidence of serious health problems, and disseminating accurate vaccine information through various channels^40, 47, 52, 53^ (posts on social media platforms, recommendations from trusted clinicians, announcements from public health organizations). Moreover, increasing public trust in the vaccine’s development and helping people see that their community is increasingly taking the vaccine (if true) could strengthen positive vaccine safety beliefs^54^. Interestingly, social responsibility and anticipated regret also increase vaccine intention indirectly by reducing vaccine safety concerns. One explanation is that people who accept social responsibility and/or anticipate regret might be motivated to justify their vaccination decisions by convincing themselves that vaccines will be safe. By indirectly encouraging self-persuasion^55^, we could potentially change the vaccination behaviour of those resistant to direct vaccine safety messages, especially if they perceive threats to their autonomy and freedom of choice^56^. In sum, interventions on social responsibility and anticipated regret can serve as complements to the more common approaches of showcasing scientific evidence and social norms in order to further reduce concerns about vaccine safety^45^.

Our study has several limitations. First, respondents’ vaccine intent was self-reported. When reporting how likely they were to take the vaccine, respondents might have overlooked barriers such as cost and transport. This could explain why most structural barriers (e.g., delayed medical care due to work schedule) and the belief that vaccines are free were not found to be significantly causal. Second, vaccine intention, the outcome variable of this study, is not a perfect proxy for vaccine uptake. Several studies^57–60^ revealed that vaccine hesitancy is prevalent even amongst those who were vaccinated for COVID-19. At the same time, according to CUBES^61^, our behavioural framework for the survey design of this study, a high vaccine intention does not necessarily lead to vaccine uptake—there could be contextual barriers such as lack of health insurance and sick paid leave^62, 63^ that could prevent individuals with high vaccine intent to get vaccinated. Third, our data was from early 2021; since then, news of new viral variants, booster recommendations^64^, and breakthrough infections^64^ could affect people’s responses. Fourth, although BN is useful in modelling confounding if the confounding variables are included in the training data set, it is still subject to potential *latent* confounders, i.e., confounding variables not captured in the data. Fifth, error could be introduced to the causal structure of the model in the periphery due to the fact that the same statistical properties of the data could be represented by multiple, similar DAGs in an equivalent class known as Completed Partially Directed Acyclic Graph^31^. Lastly, regression-informed feature selection was done to reduce data complexity; however, the choice of regression arbitrary, and more research should be conducted to test this approach.

Despite these limitations, our study supports several existing interventions, shows how they mediate one another, and provides evidence that several key drivers and causal pathways of vaccine intention may have been underemphasised. Our results also demonstrate that BN could be an effective way to explore multiple causal pathways of other complex behavioural problems. Last, a possible extension of the present study is use a mixed method approach by first inferring precise—and potentially differing—determinants of vaccine intention of subsegments of populations using BN, followed by collecting qualitative data from each subsegment to come up with effective and targeted intervention strategies, to further optimise vaccine uptake.

## Methods

### The Surgo COVID-19 vaccine survey

To collect data on a broad number of psychobehavioural factors behind COVID-19 vaccine intention beyond a narrow scope of demographic factors, we surveyed a nationally representative sample of 2747 US residents via the National Opinion Research Center (NORC) AmeriSpeak Omnibus Survey Panel from December 21, 2020 to January 4, 2021. We measured people’s vaccine intention by asking them “*When a vaccine for COVID-19 is available to you, how likely are you to take it?”*. The survey included questions on respondents’ beliefs, risk perceptions, emotions and perceived social norms, along with their demographics (Online Appendix B). Summary statistics on the survey respondents are provided in Online Appendix H.

### Surgo COVID-19 vaccine survey weighting

Panel-based sampling weights, which were computed from the inverse probability of the selection from the NORC national frame, were used to create nationally representative sampling weights for the survey data. The panel weights were also raked to external population benchmarks. The weighting was based on 7 variables: age, gender, census division, race/ethnicity, education, housing tenure, and household phone ownership status.

### Ethics statement

This questionnaire and survey study were reviewed and approved by the Salus Institutional Review Board (protocol number 02) on 30 November 2020 with an original expiration date of 29 November 2021. A renewal request was accepted on 24 November 2021 to extend the expiration date to 24 November 2022. Per NORC procedures, participation is voluntary at the time that respondents are asked to join the panel and at the time they are asked to participate in the Surgo COVID-19 Vaccine Survey. Prior to the start of the survey respondents were given information about its purpose, and they must acknowledge that they are over 18 and give their informed consent before taking the survey. No personally identifiable data were transmitted, used, or stored for this analysis in adherence to the principles of the Declaration of Helsinki. The methods in this study were performed in accordance with all relevant guidelines and regulations.

### Autonomous machine learning of the causal Bayesian network

BN represents the probabilistic conditional dependency among variables as a set of directed edges (i.e., arrows) in a DAG (Fig. 1). The representation is compact in that an outcome node may be considered as a causal node of another variable to which its arrows emanate. By tracing the arrows backwards from a given node, one may read off its upstream causes. It is with this graphical form that BN allows us to reason the system-level view of probabilistic interplay among different causal drivers in domains from disease diagnosis to biomonitoring^29, 65, 66^.

How do we identify the structure of a DAG in the first place? A more generalised approach is to learn the DAG structure of a BN automatically using a class of algorithms called causal discovery algorithms. These algorithms learn the conditional dependencies between variables from data directly, in a hypothesis-free manner^67^. While the mathematical foundations of these approaches are beyond the current scope, there are reviews that survey some common algorithms^67–69^. An important feature is that once the structure is learnt, confounders that are present in the input dataset can be easily identified as the common cause nodes (Fig. 1). Moreover, partial expert knowledge can be integrated as a prior to the structural learning. Until recently, applications of such algorithms were only feasible to problems of few variables, due to the computational complexity involved. This has changed with new research on more efficient machine learning methods, and ever-advancing computational equipment^70^. Evaluating BN network structure is described in Online Appendix I.

Algorithms for searching SCMs can be score-, constraint-, or hybrid-based. We followed previously developed proprietary procedure^33^, which is a hybrid-based implementation using the constraint-based PC algorithm as an initialization step, and the Quotient Normalized Maximum Likelihood as the score for the subsequent score-based Markov Chain Monte Carlo optimization. The computation takes about a day on an Amazon Web Service’s z1d.6xlarge EC2 instance. In structural accuracy tests using synthetic data sets, we have found this hybrid algorithms to outperform common algorithms such as PC alone that could otherwise produce results orders of magnitude faster. There may be alternative circumstances where computational speed outweighs the benefit of accuracy gains.

Although BN is useful in modelling confounding if the confounding variables are included in the training data set, it is still subject to potential latent confounders, i.e., confounding variables not captured in the data. There are algorithms that attempt to include information about potential latent confounders between pairs of nodes, most notably the Fast Causal Inference (FCI) algorithm and its variants^71, 72^. Anecdotally we have found with a complex graph and a limited dataset, FCI tends to identify latent confounders occurring everywhere in the graph. While it certainly can be true, it is not particularly helpful for practitioners.

### Bayesian network: representation and causal discovery

To infer the drivers of vaccine intention, a structural learning algorithm was used to build a BN model with 2,477 completed survey responses (out of the 2,747 total survey responses). Due to the complexity of the structural learning task, a limited number of variables (and limited number of levels for each variable) can be included in the model so that it can be learnt within a reasonable amount of time and accuracy. In addition, given the sample size of our vaccine survey datasets, there is a trade-off between the number of included variables and model performance. In particular, the unsupervised learning of where the edges should be and their directions is an NP-hard problem^73^, since the number of possible topologies grows super-exponentially with the variable included. Given just 5 variables to consider, there are 29,281 possible DAGs. Given just 10 variables, there are more than 4 quintillion possibilities. Aside from a more efficient search algorithm such as Order Markov Chain Monte Carlo, an effective way to reduce this complexity is careful selection of input variables. Using a previously developed procedure^33^, we determined that a BN with 45 variables and at most 3 discrete or ordinal levels achieves the optimal balance of model complexity and performance. For this reason, using the feature selection process (described in the section below), we selected 45 variables as inputs for the causal discovery algorithm. In addition, we converted all continuous variables into categorical/ordinal variables with at most 3 levels. For example, vaccine intention (originally on a scale of 0–10) is discretised into 3 levels: Low (0–3), Moderate (4–6) and High (7–10). The expected performance of our model was proxied by several graph metrics^33^ (Online Appendix I), including the V-structure^74^ precision, recall, and f1-score, which we found to be 0.92, 0.79, and 0.82, respectively. Note there are other approaches, including different structural learning algorithms^75^ to circumvent the complexity problem but pre-processing data is arguably the most straightforward for practitioners. Lastly, we imposed additional constraints as prior to the structure search algorithm such that obviously unreasonable relationships are precluded (e.g., worries about catching covid causes race).

### Linear regression as a feature selection process to prioritise variables to include in the BN

We began our investigation with a weighted least squares (WLS) regression model to establish correlational relationships between 68 behavioural factors and self-reported vaccine intention. The primary purpose of this model is to aid variable selection for the BN, which is the main model of this study.

We trained the WLS model with data from the Surgo COVID-19 Vaccine Survey (n = 2454 completed cases). Note that the number of complete cases that were used to train the WLS model is slightly smaller than the 2477 completed cases that were used as inputs to the causal discovery algorithm. This is because only a subset of the variables that were used to train the WLS model was used as inputs for causal discovery. The variables in the training data for the WLS model are in their original scales (e.g., respondents were asked in the survey to rate how likely they are to get the COVID-19 vaccine on a scale of 0–10; thus, *vaccine intention* has a scale of 0–10). The model is of the form:

$${Y}_{i}=a + {X}_{i}b +{u}_{i}$$

Here ***Y***_***i***_ is the self-reported vaccine intention of individual *i* (with a scale of 0–10, with 0 indicating that the respondent has extremely low intention of taking the COVID-19 vaccine, and 10 indicating extremely high intention), and ${X}_{i}$ is a vector of causal factors that might affect the individual’s vaccine intention.

The results from the regression analysis, summarised in Tables J1–J5 in Online Appendix J, usefully inform variable selection for the BN. Variables were selected for the BN based on three criteria: (1) the strength of association of the variable with vaccine intention from our regression model, (2) the amount of corroborating evidence from existing literature for the relationship between the variable and vaccine intention (or other similar outcomes), and (3) the extent to which the variable can be easily manipulated by interventions. The full list of 45 variables that were included for the BN is in Table 1 below. To ground the BN in the behavioural context, we used a behavioural framework that we had previously developed (CUBES)^61^ to assign each of the variables in the BN a corresponding causal factor category for easier interpretation (Table 1). The primary reason for this step is to reduce data complexity and the choice of linear regression is subjective for its straightforwardness; we could have chosen other procedures such as mutual information maximization to augment this process.

Some variables that were excluded from the BN (according to the criteria above) are knowledge about the COVID-19 disease, flu vaccine uptake status, feeling depressed in the past three days, and the belief that natural immunity is more effective than vaccine-induced immunity.

### Bayesian network: interventional queries

For Bayesian network, an *interventional query*, or *what-if analysis* is defined as estimating the change of outcome values as the result of changing the value of another variable (the “intervened” variable, known as the evidence variable) while holding the values of all other variables constant. This is sometimes referred to as do-calculus. We estimated the pre-intervention and post-intervention probability distribution functions for the outcome variable, and then computed the interventional odds ratio (OR). The interventional OR estimates how much more likely an outcome is given a change in an evidence variable. For more details on the definition of interventional OR, please refer to Online Appendix A.

### Comparison between multinomial logistic regression and BN

We compare the findings from the BN and a multinomial logistic regression model to highlight the differences in the conclusions that we would have drawn had we relied on the correlational relationships from the regression model instead of the estimated causal relationships from the BN.

To facilitate this comparison, we built a second multinomial logistic regression model using the same variables as for the BN—i.e., 44 causal factors that are discretised to at most 3 levels as independent variables, and vaccine intention with 3 levels (Low, Moderate, and High) as the dependent variable. To simplify our comparison, a causal factor is deemed to have significant association with vaccine intention by the multinomial logistic regression model if any of its associated multinomial logit values is significant at the 5% level. In other words, if a “significant” causal factor has two levels (e.g., Low income, High income), then either the multinomial logit estimate comparing respondents with Low income to High income for High vaccine intention relative to Low vaccine intention is significant, or the estimate comparing respondents with Low income to High income for High vaccine intention relative to Moderate vaccine intention is significant. Similarly, in the BN, a causal factor is deemed to have a significant estimated causal effect on vaccine intention if any of its intervention OR is significant. Using these definitions of “significance”, we determined and compared the significant drivers from the regression model and the BN.

## Supplementary Information

Supplementary Information.

## Data Availability

The Surgo COVID-19 Vaccine Survey data that have been used for the present study are not publicly available but are available upon request. Please contact Dr. Sema K. Sgaier at semasgaier@surgohealth.com.
