# Supplementary material for: Discovery of interconnected causal drivers of COVID-19 vaccination intentions in the US using a causal Bayesian network
Source: Sci Rep. 2023 May 16;13:6988. doi: 10.1038/s41598-023-33745-4 (PMC10188432; doi:10.1038/s41598-023-33745-4)
Supplement: Supplementary file 1 — Supplementary Information. [file 41598_2023_33745_MOESM1_ESM.docx]

**Supplementary Information for: Discovery of causal drivers of COVID-19 vaccination intentions in the US using causal Bayesian Network**

**Authors: Henry Fung^1,2^***,* **Sema K. Sgaier^1,2,3^** *,***Vincent S. Huang^1,2^**

^1^ *Surgo Health, Washington, DC, USA*

^2^ *Surgo Ventures, Washington, DC, USA*

^3^ *Department of Global Health, University of Washington, Seattle, Washington, USA*

*Corresponding author: Sema K. Sgaier semasgaier@surgohealth.com*

[Appendix A: Interventional Performance Calculation for Causal Bayesian Network 1](#_Toc908958447)

[Appendix B: The Surgo COVID-19 Vaccine Survey 2](#_Toc149567307)

[Appendix C: Comparing the findings from BN and a multinomial logistic regression model 3](#_Toc1903782637)

[Appendix D: Interventional Queries on the Learnt BN 22](#_Toc1092933879)

[Appendix E: Interventional Queries on Causes of Cause 42](#_Toc810097698)

[Appendix F: Weighted Least Square models with variables related to political affiliation 59](#_Toc582642845)

[Appendix G: Using the BN to infer the impact of political affiliation 60](#_Toc437636520)

[Appendix H: Survey respondent characteristics 63](#_Toc1548378563)

[Appendix I: Estimated BN Performance 64](#_Toc2081140469)

[Appendix J: Regression Analyses for Selecting Features for the Bayesian Network (BN) 65](#_Toc987492816)

# **Appendix A: Interventional Performance Calculation for Causal Bayesian Network**

One of the key uses of a causal Bayesian Network model is that, for a given outcome variable of interest, one can test hypothetical interventions on each variable. One can then compute the interventional odds ratio (OR) of how the outcome may change based on the intervention.


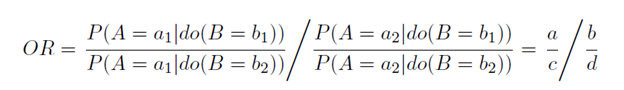


The results of this “intervention” encompass both the causal structure learned, and the parameters (estimated by Maximum Likelihood) of the conditional probability tables at each variable. We calculate the standard error for the odds ratios by:


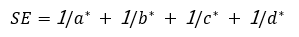


Where a* is the frequency obtained from the training data for term a; P(A=a_1_ | do(B=b_1_))

95% confidence intervals are then obtained by:


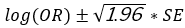


# **Appendix B:** **The Surgo COVID-19 Vaccine Survey**

We surveyed a nationally representative sample of 2,747 US residents via the National Opinion Research Center (NORC) AmeriSpeak Omnibus Survey Panel from December 21, 2020 to January 4, 2021. We measured people’s vaccine intention by asking them “*When a vaccine for COVID-19 is available to you, how likely are you to take it?”*. We designed the Surgo COVID-19 Vaccine Survey using the CUBES behavioral framework^52^. Respondents were screen for basic demographics information (Appendix H). The survey took 45 minutes and was intended to elicit a comprehensive picture of the potential barriers to and enablers of vaccination intention. In addition to sociodemographic factors, these include:

- Structural barriers and enablers of COVID-19 vaccination (e.g. access to health care)
- Beliefs and perceptions about the COVID-19 pandemic and COVID-19 vaccines (e.g., the nature and status of the pandemic, conspiracy theories, perception of the effectiveness of vaccine)
- Influencers and sources of information (e.g., media source, health authorities, and political affiliation)
- Perceived risk of getting COVID-19
- Perceived risk of side effects from COVID-19 vaccine
- Social norms on COVID-19 vaccination (e.g., perception of what the respondent’s community response to the pandemic)
- Health-seeking and previous vaccination behaviors (e.g., flu vaccine)
- Knowledge about COVID-19 vaccine and the COVID-19 pandemic

Respondents gave informed consent prior to taking the survey and was informed that they could stop at any time. The survey instrument is currently proprietary. However, access for non-commercial use can be granted from the authors on a per-request basis.

# **Appendix C: Comparing the findings from BN and a multinomial logistic regression model**

We present a detailed comparison between the findings from the BN and a multinomial logistic regression model (Tables C1-C2).

Both models were trained using the same dataset with 45 categorical/ordinal variables. Behavioural correlates that have a significant association with vaccine intention (estimated by the regression model) are compared with behavioural drivers that have a significant interventional OR (estimated from the BN) for potential targets for interventions to increase vaccine uptake. A comparison of the two models is summarized in Table C3.

Although both models identify *social responsibility, the belief that COVID vaccines will be unsafe*, and *anticipated regret* as important determinants of vaccine intention, there are also notable differences in the findings. First, the regression model identifies *the belief that a tracking chip is implanted in me through vaccine*, *the expected chance of getting long-term side-effects* *from COVID vaccines* and *the* *reference community expects me to take COVID-19 vaccine* as determinants of vaccine intention; however, these drivers do not have significant effects in the BN. Second, the regression model identifies belief in several sources of COVID-19 information – notably from public health organizations – as having significant associations with vaccine intention. In contrast, while the beliefs in various information sources are causal factors, their effects were not found to be significant in the BN. Third, there are discrepancies in the type of vaccine safety concerns that matter for vaccine intention. The BN suggests that *trust in the vaccine development process* is an important concern, while the regression model identifies *the belief that COVID-19 vaccine is tested for the safety of my race* and *the belief that COVID-19 testing is too rushed* as important concerns.

Tables C1-C2 present the multinomial logistic outputs. The independent variables are the 44 behavioural drivers in the BN, and the dependent variable is vaccine intention (discretized into 3 levels: Low, Moderate, and High).

**Table C1: Multinomial logistic regression outputs with low vaccine intention as the reference level for the dependent variable, and high vaccine intention as the target level for the dependent variable**

| **Variable** | **Regression coefficients** | **p-value** | **lower 95% confidence interval** | **upper 95% confidence interval** |
| --- | --- | --- | --- | --- |
| **Behaviours** | | | | |
| seek check-up if I have symptoms in general: Yes (vs. No) | 0.764 | 0.362 | 0.424 | 1.375 |
| willing to take the COVID-19 vaccine in the first three months: Unsure (vs. No) | 0.606** | 0.032 | 0.383 | 0.959 |
| willing to take the COVID-19 vaccine in the first three months: Yes (vs. No) | 290.302*** | 0.000 | 34.877 | 2416.364 |
| **Beliefs and perceptions** | | | | |
| believes that a tracking chip is implanted in me through vaccine: Yes (vs. No) | 0.254*** | 0.001 | 0.112 | 0.575 |
| COVID-19 is perceived to be dangerous to my community: Yes (vs. No) | 0.804 | 0.390 | 0.485 | 1.334 |
| believes that COVID-19 testing is too rushed: Yes (vs. No) | 0.278*** | 0.000 | 0.174 | 0.443 |
| believes COVID-19 vaccine is free: Yes (vs. No) | 0.948 | 0.813 | 0.582 | 1.543 |
| believes COVID vaccine will be unsafe: Yes (vs. No) | 0.428** | 0.016 | 0.214 | 0.855 |
| believes people of my race are fairly treated in a health-care setting: Yes (vs. No) | 1.526 | 0.169 | 0.832 | 2.800 |
| believes in having responsibility to get vaccinated for COVID-19 to protect others: Yes (vs. No) | 8.019*** | 0.000 | 4.528 | 14.199 |
| has control over getting COVID: Yes (vs. No) | 0.996 | 0.967 | 0.629 | 1.577 |
| believes COVID-19 vaccine is tested for the safety and effectiveness of my race: Agree (vs. Disagree) | 2.861*** | 0.001 | 1.497 | 5.469 |
| believes COVID-19 vaccine is tested for the safety and effectiveness of my race: Neither agree or disagree (vs. Disagree) | 2.125*** | 0.010 | 1.200 | 3.762 |
| trust in vaccine development process of pharmaceutical firms: Agree (vs. Disagree) | 1.279 | 0.368 | 0.742 | 2.204 |
| **Demographics** | | | | |
| age: 35-64 (vs. 18-34) | 1.136 | 0.604 | 0.690 | 1.869 |
| age: 65 or over (vs. 18-34) | 2.072* | 0.056 | 0.977 | 4.394 |
| education: Bachelor's degree or more (vs. High school or less) | 1.214 | 0.496 | 0.685 | 2.152 |
| education: Some college or associates (vs. High school or less) | 1.135 | 0.620 | 0.675 | 1.911 |
| gender: Woman (vs. Man) | 1.683** | 0.019 | 1.087 | 2.606 |
| income: $30,000 to under $100,000 (vs. $100,000 or more) | 0.838 | 0.539 | 0.469 | 1.497 |
| income: Less than $30,000 (vs. $100,000 or more) | 0.607 | 0.137 | 0.313 | 1.177 |
| is essential worker: Yes (vs. No) | 1.186 | 0.497 | 0.717 | 1.961 |
| political affiliation: Democrat (vs. Republican) | 1.431 | 0.283 | 0.738 | 2.774 |
| political affiliation: Independent or other (vs. Republican) | 0.830 | 0.501 | 0.477 | 1.446 |
| race: Black (vs. White) | 0.941 | 0.852 | 0.455 | 1.946 |
| race: Other minorities (vs. White) | 1.121 | 0.652 | 0.668 | 1.882 |
| urbanicity: Rural (vs. Urban) | 0.749 | 0.329 | 0.416 | 1.349 |
| **Emotions** | | | | |
| regret if I did not take COVID vaccine: Yes (vs. No) | 3.858*** | 0.000 | 2.342 | 6.355 |
| worries about catching COVID-19: A great deal (vs. Not at all or not much) | 1.997* | 0.083 | 0.909 | 4.385 |
| worries about catching COVID-19: Moderate (vs. Not at all or not much) | 2.097*** | 0.005 | 1.250 | 3.518 |
| **Influencers/channels** | | | | |
| gets information on COVID-19 from Fox News: Yes (vs. No) | 0.665* | 0.087 | 0.416 | 1.064 |
| gets information on COVID-19 from left-wing media: Yes (vs. No) | 0.773 | 0.291 | 0.477 | 1.253 |
| gets information on COVID-19 from scientists: Yes (vs. No) | 0.982 | 0.923 | 0.599 | 1.610 |
| gets information on COVID-19 from social media: Yes (vs. No) | 0.602* | 0.060 | 0.354 | 1.023 |
| believes in COVID-19 information from public health organizations: Agree (vs. Disagree) | 2.344** | 0.039 | 1.043 | 5.270 |
| believes in COVID-19 information from public health organizations: Sometimes agree or disagree (vs. Disagree) | 1.089 | 0.809 | 0.510 | 2.324 |
| believes in COVID-19 information from my local community: Agree (vs. Disagree) | 0.448* | 0.070 | 0.187 | 1.073 |
| believes in COVID-19 information from my local community:  Sometimes agree or disagree (vs. Disagree) | 0.503** | 0.036 | 0.264 | 0.959 |
| believes in COVID-19 information from my physician: Agree (vs. Disagree) | 2.173 | 0.182 | 0.689 | 6.860 |
| believes in COVID-19 information from my physician: Sometimes agree or disagree (vs. Disagree) | 2.411 | 0.134 | 0.757 | 7.678 |
| believes in COVID-19 information from my social media contacts: Agree (vs. Disagree) | 1.683 | 0.288 | 0.636 | 4.454 |
| believes in COVID-19 information from my social media contacts: Sometimes agree or disagree (vs. Disagree) | 1.558 | 0.130 | 0.874 | 2.777 |
| believes in COVID-19 information from Trump: Agree (vs. Disagree) | 0.595 | 0.140 | 0.298 | 1.190 |
| believes in COVID-19 information from Trump: Sometimes agree or disagree (vs. Disagree) | 0.593* | 0.057 | 0.345 | 1.019 |
| **Outcome expectation** | | | | |
| expected chance of dying from COVID with no vaccine: High or almost certain (vs. Almost zero or low) | 0.322** | 0.024 | 0.120 | 0.866 |
| expected chance of dying from COVID with no vaccine: Moderate (vs. Almost zero or low) | 0.474** | 0.025 | 0.247 | 0.911 |
| expected chance of getting COVID with no vaccine: High or almost certain (vs. Almost zero or low) | 2.336** | 0.047 | 1.008 | 5.414 |
| expected chance of getting COVID with no vaccine: Moderate (vs. Almost zero or low) | 2.263** | 0.010 | 1.216 | 4.212 |
| expected chance of getting seriously ill from COVID-19: High or almost certain (vs. Almost zero or low) | 3.962*** | 0.008 | 1.419 | 11.062 |
| expected chance of getting seriously ill from COVID-19: Moderate (vs. Almost zero or low) | 2.339** | 0.018 | 1.157 | 4.728 |
| expected chance of getting long-term side-effects from COVID vaccine: High or almost certain (vs. Almost zero or low) | 0.264*** | 0.000 | 0.133 | 0.524 |
| expected chance of getting long-term side-effects from COVID vaccine: Moderate (vs. Almost zero or low) | 0.529** | 0.019 | 0.310 | 0.903 |
| **Social influences** | | | | |
| proportion of community that I think will take the COVID-19 vaccine: More than half or all (vs. None or fewer than half) | 2.660*** | 0.000 | 1.662 | 4.257 |
| reference community**** expects me to take COVID-19 vaccine: Not sure or no answer (vs. No) | 3.480*** | 0.000 | 1.958 | 6.187 |
| reference community expects me to take COVID-19 vaccine: Yes (vs. No) | 2.791*** | 0.000 | 1.579 | 4.932 |
| reference community considers COVID-19 a serious threat: Not sure (vs. No) | 1.610 | 0.278 | 0.674 | 3.846 |
| reference community considers COVID-19 a serious threat: Yes (vs. No) | 1.991** | 0.032 | 1.060 | 3.738 |
| **Structural enablers/barriers** | | | | |
| delayed medical care due to childcare: Yes (vs. No) | 1.258 | 0.599 | 0.519 | 3.052 |
| delayed medical care due to cost: Yes (vs. No) | 1.070 | 0.783 | 0.635 | 1.803 |
| delayed medical care due to work schedule: Yes (vs. No) | 0.993 | 0.960 | 0.556 | 1.773 |
| Easy to get COVID-19 vaccine once it is available: Yes (vs. No) | 1.334 | 0.205 | 0.851 | 2.091 |
| has health insurance: Yes (vs. No) | 1.929** | 0.049 | 0.999 | 3.726 |
| have personal care physician: Yes (vs. No) | 0.531** | 0.033 | 0.296 | 0.952 |
| **Observations**  **Residual Deviance: 2046.984**  **AIC** | **2477**  **2046.98**  **2302.98** |  |  |  |

*Note:* *p<0.1; **p<0.05; ***p<0.01

**** respondents mostly specified their immediate family and friends as their reference community

**Table C2: Multinomial logistic regression outputs with moderate vaccine intention as the reference level for the dependent variable, and high vaccine intention as the target level for the dependent variable**

| **Variable** | **Regression coefficients** | **p-value** | **lower 95% confidence interval** | **upper 95% confidence interval** |
| --- | --- | --- | --- | --- |
| **Behaviours** | | | | |
| seek check-up if I have symptoms in general: Yes (vs. No) | 1.216 | 0.408 | 0.758 | 1.951 |
| willing to take the COVID-19 vaccine in the first three months: Unsure (vs. No) | 1.951*** | 0.001 | 1.336 | 2.849 |
| willing to take the COVID-19 vaccine in the first three months: Yes (vs. No) | 21.017*** | 0.000 | 12.867 | 34.329 |
| **Beliefs and perceptions** | | | | |
| **believes that a tracking chip is implanted in me through vaccine: Yes (vs. No)** | **0.363***** | **0.006** | **0.176** | **0.747** |
| COVID-19 is perceived to be dangerous to my community: Yes (vs. No) | 1.133 | 0.514 | 0.771 | 1.667 |
| believes that COVID-19 testing is too rushed: Yes (vs. No) | 0.397*** | 0.000 | 0.275 | 0.575 |
| believes COVID-19 vaccine is free: Yes (vs. No) | 0.897 | 0.554 | 0.621 | 1.297 |
| believes COVID vaccine will be unsafe: Yes (vs. No) | 1.080 | 0.798 | 0.570 | 2.047 |
| believes people of my race are fairly treated in a health-care setting: Yes (vs. No) | 1.056 | 0.818 | 0.634 | 1.759 |
| believes in having responsibility to get vaccinated for COVID-19 to protect others: Yes (vs. No) | 3.524*** | 0.000 | 2.411 | 5.152 |
| has control over getting COVID: Yes (vs. No) | 0.948 | 0.764 | 0.654 | 1.375 |
| believes COVID-19 vaccine is tested for the safety and effectiveness of my race: Agree (vs. Disagree) | 1.537* | 0.098 | 0.921 | 2.565 |
| believes COVID-19 vaccine is tested for the safety and effectiveness of my race: Neither agree or disagree (vs. Disagree) | 1.386 | 0.184 | 0.853 | 2.253 |
| trust in vaccine development process of pharmaceutical firms: Agree (vs. Disagree) | 0.809 | 0.384 | 0.499 | 1.314 |
| **Demographics** | | | | |
| age: 35-64 (vs. 18-34) | 1.603** | 0.018 | 1.082 | 2.375 |
| age: 65 or over (vs. 18-34) | 3.382*** | 0.000 | 1.917 | 5.968 |
| education: Bachelor's degree or more (vs. High school or less) | 1.141 | 0.541 | 0.738 | 1.763 |
| education: Some college or associates (vs. High school or less) | 1.126 | 0.569 | 0.740 | 1.714 |
| gender: Woman (vs. Man) | 1.489** | 0.021 | 1.060 | 2.090 |
| income: $30,000 to under $100,000 (vs. $100,000 or more) | 1.049 | 0.806 | 0.689 | 1.599 |
| income: Less than $30,000 (vs. $100,000 or more) | 0.876 | 0.584 | 0.537 | 1.430 |
| is essential worker: Yes (vs. No) | 1.246 | 0.277 | 0.834 | 1.862 |
| political affiliation: Democrat (vs. Republican) | 1.158 | 0.551 | 0.704 | 1.905 |
| political affiliation: Independent or other (vs. Republican) | 0.652* | 0.054 | 0.420 | 1.010 |
| race: Black (vs. White) | 1.118 | 0.695 | 0.622 | 2.009 |
| race: Other minorities (vs. White) | 1.113 | 0.586 | 0.747 | 1.658 |
| urbanicity: Rural (vs. Urban) | 0.699 | 0.137 | 0.435 | 1.125 |
| **Emotions** | | | | |
| regret if I did not take COVID vaccine: Yes (vs. No) | 1.146 | 0.496 | 0.767 | 1.710 |
| worries about catching COVID-19: A great deal (vs. Not at all or not much) | 1.308 | 0.371 | 0.719 | 2.377 |
| worries about catching COVID-19: Moderate (vs. Not at all or not much) | 1.248 | 0.278 | 0.832 | 1.869 |
| **Influencers/channels** | | | | |
| gets information on COVID-19 from Fox News: Yes (vs. No) | 0.715* | 0.076 | 0.492 | 1.038 |
| gets information on COVID-19 from left-wing media: Yes (vs. No) | 0.797 | 0.226 | 0.551 | 1.154 |
| gets information on COVID-19 from scientists: Yes (vs. No) | 1.115 | 0.557 | 0.768 | 1.617 |
| gets information on COVID-19 from social media: Yes (vs. No) | 0.751 | 0.188 | 0.489 | 1.154 |
| believes in COVID-19 information from public health organizations: Agree (vs. Disagree) | 1.302 | 0.443 | 0.655 | 2.588 |
| believes in COVID-19 information from public health organizations: Sometimes agree or disagree (vs. Disagree) | 0.791 | 0.481 | 0.406 | 1.540 |
| believes in COVID-19 information from my local community: Agree (vs. Disagree) | 0.443** | 0.014 | 0.232 | 0.848 |
| believes in COVID-19 information from my local community: Sometimes agree or disagree (vs. Disagree) | 0.529** | 0.016 | 0.315 | 0.888 |
| believes in COVID-19 information from my physician: Agree (vs. Disagree) | 1.618 | 0.347 | 0.585 | 4.474 |
| believes in COVID-19 information from my physician: Sometimes agree or disagree (vs. Disagree) | 1.784 | 0.264 | 0.638 | 4.988 |
| believes in COVID-19 information from my social media contacts: Agree (vs. Disagree) | 1.005 | 0.968 | 0.505 | 2.002 |
| believes in COVID-19 information from my social media contacts: Sometimes agree or disagree (vs. Disagree) | 1.455* | 0.095 | 0.935 | 2.265 |
| believes in COVID-19 information from Trump: Agree (vs. Disagree) | 0.708 | 0.203 | 0.413 | 1.212 |
| believes in COVID-19 information from Trump: Sometimes agree or disagree (vs. Disagree) | 0.664** | 0.048 | 0.441 | 0.997 |
| **Outcome expectation** | | | | |
| expected chance of dying from COVID with no vaccine: High or almost certain (vs. Almost zero or low) | 0.673 | 0.289 | 0.320 | 1.412 |
| expected chance of dying from COVID with no vaccine: Moderate (vs. Almost zero or low) | 0.965 | 0.868 | 0.595 | 1.567 |
| expected chance of getting COVID with no vaccine: High or almost certain (vs. Almost zero or low) | 1.870* | 0.059 | 0.972 | 3.597 |
| expected chance of getting COVID with no vaccine: Moderate (vs. Almost zero or low) | 1.115 | 0.654 | 0.677 | 1.837 |
| expected chance of getting seriously ill from COVID-19: High or almost certain (vs. Almost zero or low) | 2.125* | 0.055 | 0.981 | 4.601 |
| expected chance of getting seriously ill from COVID-19: Moderate (vs. Almost zero or low) | 1.570* | 0.094 | 0.923 | 2.668 |
| expected chance of getting long-term side-effects from COVID vaccine: High or almost certain (vs. Almost zero or low) | 0.538** | 0.034 | 0.303 | 0.957 |
| expected chance of getting long-term side-effects from COVID vaccine: Moderate (vs. Almost zero or low) | 0.597** | 0.010 | 0.402 | 0.886 |

| **Social influences** | | | | |
| --- | --- | --- | --- | --- |
| proportion of community that I think will take the COVID-19 vaccine: More than half or all (vs. None or fewer than half) | 2.079*** | 0.000 | 1.418 | 3.049 |
| reference community**** expects me to take COVID-19 vaccine: Not sure or no answer (vs. No) | 2.176*** | 0.001 | 1.363 | 3.472 |
| reference community expects me to take COVID-19 vaccine: Yes (vs. No) | 1.974*** | 0.002 | 1.275 | 3.057 |
| reference community considers COVID-19 a serious threat: Not sure (vs. No) | 1.004 | 0.971 | 0.488 | 2.066 |
| reference community considers COVID-19 a serious threat: Yes (vs. No) | 1.199 | 0.491 | 0.707 | 2.036 |
| **Structural enablers/barriers** | | | | |
| delayed medical care due to childcare: Yes (vs. No) | 2.782** | 0.011 | 1.262 | 6.134 |
| delayed medical care due to cost: Yes (vs. No) | 1.170 | 0.454 | 0.770 | 1.779 |
| delayed medical care due to work schedule: Yes (vs. No) | 0.806 | 0.341 | 0.513 | 1.265 |
| Easy to get COVID-19 vaccine once it is available: Yes (vs. No) | 1.109 | 0.541 | 0.788 | 1.561 |
| has health insurance: Yes (vs. No) | 1.263 | 0.387 | 0.738 | 2.161 |
| have personal care physician: Yes (vs. No) | 0.443*** | 0.001 | 0.273 | 0.721 |
| **Observations**  **Residual Deviance**  **AIC** | **2477**  **2046.79**  **2302.79** |  |  |  |

*Note:* *p<0.1; **p<0.05; ***p<0.01

**** respondents mostly specified their immediate family and friends as their reference community

**Table C3:** A comparison of findings from BN and multinomial logistic regression. “**X***” indicates that the variable has a significant association with vaccine intention (for the multinomial logistic regression), or vaccine intention is significantly affected by a hypothetical intervention on the variable (for the BN). For the BN, “X” indicates that the variable is identified as an upstream factor of vaccine intention by the causal discovery algorithm but changing the variable value itself would not have a significant effect on vaccine intention. Variables with no indicators are either not significantly associated with vaccine intention (for the regression model) or non-causal to vaccine intention (for the BN). The multinomial logistic regression model contains the same variables that were used in the BN: 44 behavioural drivers as independent variables and vaccine intention as the dependent variable. No interaction terms were assumed. All variables are discretized to have at most three levels of measurement.

| **Variable** | **Correlates of vaccine intention identified by Multinomial Logistic Regression** | **Drivers of vaccine intention identified by the BN** | **Behavioural driver category** |
| --- | --- | --- | --- |
| seek check-up if I have symptoms in general |  | X | behaviours |
| willing to take the COVID-19 vaccine in the first three months (early adopter) | **X*** | **X*** | intentions |
| believes that a tracking chip is implanted in me through vaccine | **X*** |  | beliefs and perceptions |
| COVID-19 is perceived to be dangerous to my community |  | **X*** | beliefs and perceptions |
| believes that COVID-19 vaccine testing is too rushed | **X*** |  | beliefs and perceptions |
| believes COVID-19 vaccine is free |  |  | beliefs and perceptions |
| believes that COVID vaccine will be unsafe | **X*** | **X*** | beliefs and perceptions |
| believes people of my race are fairly treated in a health-care setting |  |  | beliefs and perceptions |
| believes in having responsibility to get vaccinated for COVID-19 to protect others (social responsibility) | **X*** | **X*** | beliefs and perceptions |
| believes in having control over getting COVID-19 |  | X | beliefs and perceptions |
| believes COVID-19 vaccine is tested for the safety and effectiveness of my race | **X*** |  | beliefs and perceptions |
| trust in vaccine development process of pharmaceutical firms |  | **X*** | beliefs and perceptions |
| age | **X*** | X | demographics |
| education |  | X | demographics |
| gender | **X*** | X | demographics |
| income |  |  | demographics |
| is essential worker |  | X | demographics |
| political affiliation |  | **X*** | demographics |
| race |  | X | demographics |
| urbanicity |  | X | demographics |
| regret if I did not take COVID vaccine and got COVID (anticipated regret) | **X*** | **X*** | emotions |
| worries about catching COVID-19 | **X*** | **X*** | emotions |
| gets information on COVID-19 from Fox News |  |  | influencers/channels |
| gets information on COVID-19 from left-wing media |  |  | influencers/channels |
| gets information on COVID-19 from scientists |  | X | influencers/channels |
| gets information on COVID-19 from social media |  |  | influencers/channels |
| believes in COVID-19 information from public health organizations | **X*** |  | influencers/channels |
| believes in COVID-19 information from my local community | **X*** | X | influencers/channels |
| believes in COVID-19 information from my physician |  |  | influencers/channels |
| believes in COVID-19 information from my social media contacts |  | X | influencers/channels |
| believes in COVID-19 information from President Trump | **X*** | X | influencers/channels |
| expected chance of dying from COVID with no vaccine | **X*** | X | outcome expectation |
| expected chance of getting COVID with no vaccine | **X*** | **X*** | outcome expectation |
| expected chance of getting seriously ill from COVID-19 | **X*** | X | outcome expectation |
| expected chance of getting long-term side-effects from COVID vaccine | **X*** |  | outcome expectation |
| proportion of community that I think will take the COVID-19 vaccine | **X*** | **X*** | social influences |
| reference community** expects me to take COVID-19 vaccine | **X*** | X | social influences |
| reference community considers COVID-19 a serious threat | **X*** |  | social influences |
| delayed medical care due to childcare | **X*** | **X*** | structural enablers/barriers |
| delayed medical care due to cost |  |  | structural enablers/barriers |
| delayed medical care due to work schedule |  | X | structural enablers/barriers |
| believes that it is easy to get COVID-19 vaccine once it is available |  | X | structural enablers/barriers |
| has primary care physician | **X*** | X | structural enablers/barriers |
| has health insurance | **X*** | X | structural enablers/barriers |
| **Number of variables that are suggested by the model to have significant effect on vaccine intention** | **22** | **11** |  |

*Note:* ** respondents mostly specified their immediate family and friends as their reference community

# **Appendix D: Interventional Queries on the Learnt BN**


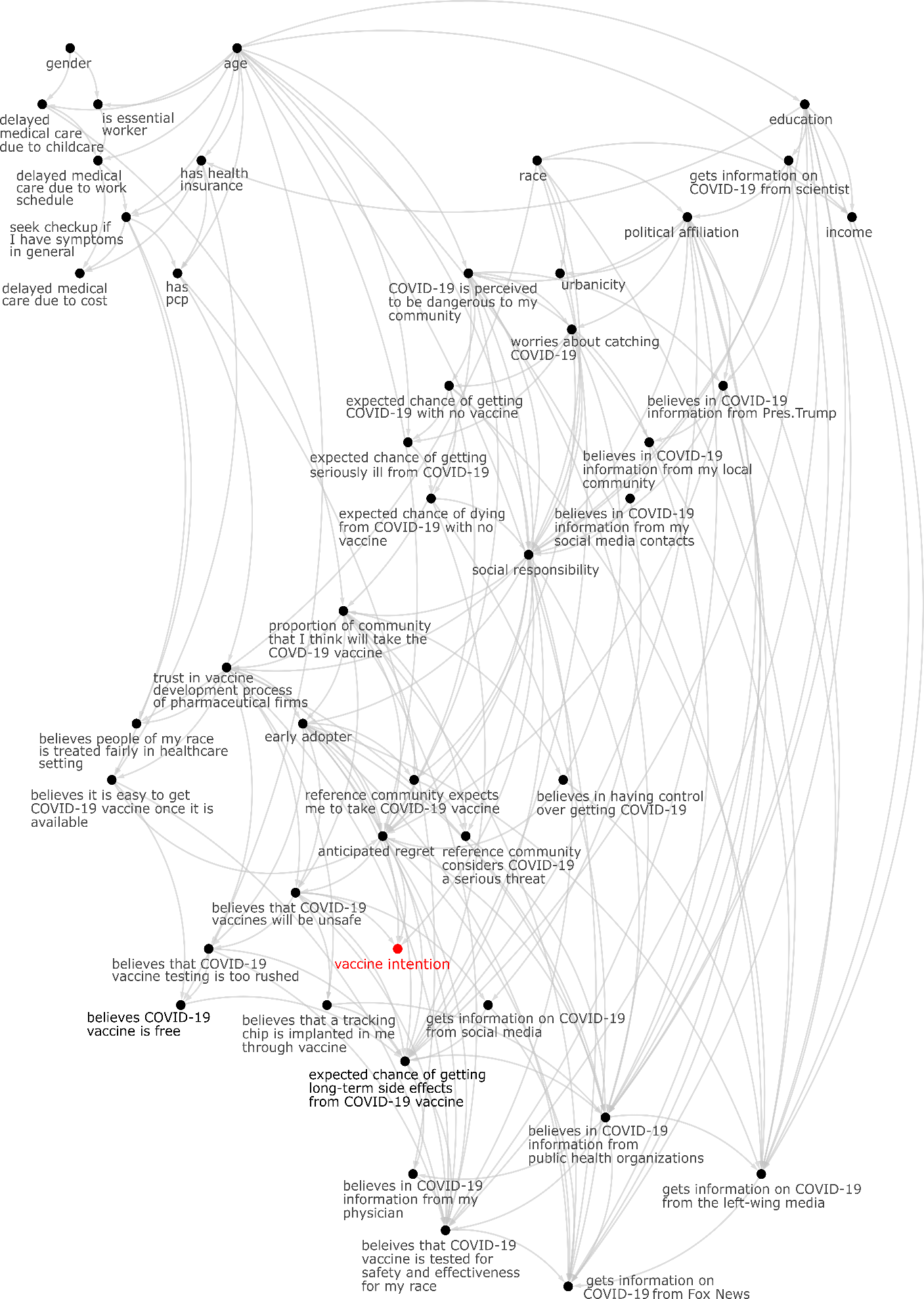


**Figure D1:** The BN learnt from Surgo COVID-19 Vaccine Survey data. All direct and indirect causes and non-causes of vaccine intention are included. The red node and text indicate the outcome variable: vaccine intention.

This section presents the interventional queries that undergird the results in the main paper.

Tables D1-D7 present interventional odds ratios (OR) – the ratio of odds of **high** vaccine intention and **low** vaccine intention when an intervention is deployed or not. In general, interventional OR is a relative measure of effect of changes in a certain evidence variable (in our case, the potential determinants of vaccine intention). Interventional ORs are computed from the results of interventional queries that are performed on the BN model learnt from the Surgo COVID-19 Vaccine Survey data.

**Table D1: The estimated effect of beliefs and perception on COVID-19 vaccine intention (from low intention to high intention) from interventional query on the learnt Bayesian network model**

| **Evidence (intervened) variable** | **Evidence variable reference level** | **Evidence variable target level** | **Interventional odds ratio** | **Lower 95% confidence interval** | **Upper 95% confidence interval** |
| --- | --- | --- | --- | --- | --- |
| believes COVID-19 vaccine is free | No | Yes | 1.00 | 0.85 | 1.17 |
| believes COVID-19 vaccine is tested for the safety and effectiveness of my race | Disagree | Agree | 1.00 | 0.87 | 1.15 |
| believes COVID-19 vaccine is tested for the safety and effectiveness of my race | Disagree | Neither agree or disagree | 1.00 | 0.87 | 1.15 |
| believes people of my race are fairly treated in a health-care setting | No | Yes | 1.00 | 0.86 | 1.16 |
| trust in vaccine development process of pharmaceutical firms | Disagree | Agree | 2.24* | 1.93 | 2.60 |
| believes COVID vaccine will be unsafe | Yes | No | 2.94 * | 2.56 | 3.45 |
| believes that COVID-19 testing is too rushed | No | Yes | 1.00 | 0.87 | 1.15 |
| believes in having responsibility to get vaccinated for COVID-19 to protect others | No | Yes | 49.33* | 38.64 | 62.99 |
| COVID-19 is perceived to be dangerous to my community | No | Yes | 1.42* | 1.21 | 1.66 |
| believes that a tracking chip is implanted in me through vaccine | No | Yes | 1.00 | 0.87 | 1.15 |
| has control over getting COVID | No | Yes | 1.02 | 0.87 | 1.19 |

*Note:* * interventional odds ratio is significantly different from zero at the 5% level

**Table D2: The estimated effect of outcome expectations on COVID-19 vaccine intention (from low intention to high intention) from interventional query on the learnt Bayesian network model**

| **Evidence (intervened) variable** | **Evidence variable reference level** | **Evidence variable target level** | **Interventional odds ratio** | **Lower 95% confidence interval** | **Upper 95% confidence interval** |
| --- | --- | --- | --- | --- | --- |
| expected chance of getting seriously ill from COVID-19 | Low | Moderate | 1.00 | 0.86 | 1.17 |
| expected chance of getting seriously ill from COVID-19 | Low | High | 1.02 | 0.87 | 1.19 |
| expected chance of getting COVID with no vaccine | Low | Moderate | 1.10 | 0.94 | 1.28 |
| expected chance of getting COVID with no vaccine | Low | High | 1.17 | 1.00 | 1.37 |
| expected chance of dying from COVID with no vaccine | Low | Moderate | 1.00 | 0.86 | 1.17 |
| expected chance of dying from COVID with no vaccine | Low | High | 1.02 | 0.87 | 1.19 |
| expected chance of getting long-term side-effects from COVID vaccine | Low | Moderate | 1.00 | 0.87 | 1.15 |
| expected chance of getting long-term side-effects from COVID vaccine | Low | High | 1.00 | 0.87 | 1.15 |

*Note:* * interventional odds ratio is significantly different from zero at the 5% level

**Table D3: The estimated effect of demographic factors on COVID-19 vaccine intention (from low intention to high intention) from interventional query on the learnt Bayesian network model**

| **Evidence (intervened) variable** | **Evidence variable reference level** | **Evidence variable target level** | **Interventional odds ratio** | **Lower 95% confidence interval** | **Upper 95% confidence interval** |
| --- | --- | --- | --- | --- | --- |
| gender | Man | Woman | 1.01 | 0.86 | 1.18 |
| race | White | Black | 1.00 | 0.85 | 1.17 |
| race | White | Other minorities | 1.00 | 0.85 | 1.17 |
| political affiliation | Republican | Democrat | 1.26* | 1.08 | 1.48 |
| political affiliation | Republican | Independent | 1.06 | 0.91 | 1.24 |
| education | High school or less | Some college or associates | 1.05 | 0.89 | 1.22 |
| education | High school or less | Bachelor's degree or more | 1.06 | 0.91 | 1.25 |
| age | 18-34 | 35-64 | 1.07 | 0.92 | 1.25 |
| age | 18-34 | 65 or over | 1.14 | 0.98 | 1.34 |
| is essential worker | No | Yes | 1.00 | 0.85 | 1.17 |
| urbanicity | Urban | Rural | 0.99 | 0.85 | 1.16 |
| income | Less than $30,000 | $30,000 to under $100,000 | 1.00 | 0.85 | 1.17 |
| income | Less than $30,000 | $100,000 or more | 1.00 | 0.85 | 1.17 |

*Note:* * interventional odds ratio is significantly different from zero at the 5% level

**Table D4: The estimated effect of influencers on COVID-19 vaccine intention (from low intention to high intention) from interventional query on the learnt Bayesian network model**

| **Evidence (intervened) variable** | **Evidence variable reference level** | **Evidence variable target level** | **Interventional odds ratio** | **Lower 95% confidence interval** | **Upper 95% confidence interval** |
| --- | --- | --- | --- | --- | --- |
| believes in COVID-19 information from Trump | Disagree | Agree | 0.91 | 0.78 | 1.07 |
| believes in COVID-19 information from Trump | Disagree | Sometimes agree or disagree | 0.92 | 0.79 | 1.08 |
| believes in COVID-19 information from public health organizations | Disagree | Agree | 1.00 | 0.86 | 1.17 |
| believes in COVID-19 information from public health organizations | Disagree | Sometimes agree or disagree | 1.00 | 0.86 | 1.17 |
| believes in COVID-19 information from my physician | Disagree | Agree | 1.00 | 0.85 | 1.17 |
| believes in COVID-19 information from my physician | Disagree | Sometimes agree or disagree | 1.00 | 0.85 | 1.17 |
| believes in COVID-19 information from my local community | Disagree | Agree | 1.00 | 0.85 | 1.16 |
| believes in COVID-19 information from my local community | Disagree | Sometimes agree or disagree | 1.00 | 0.86 | 1.17 |
| believes in COVID-19 information from my social media contacts | Disagree | Agree | 1.01 | 0.87 | 1.19 |
| believes in COVID-19 information from my social media contacts | Disagree | Sometimes agree or disagree | 0.99 | 0.85 | 1.16 |
| gets information on COVID-19 from left-wing media | No | Yes | 1.00 | 0.86 | 1.17 |
| gets information on COVID-19 from Fox News | No | Yes | 1.00 | 0.86 | 1.17 |
| gets information on COVID-19 from social media | No | Yes | 1.00 | 0.87 | 1.15 |
| gets information on COVID-19 from scientists | No | Yes | 1.08 | 0.92 | 1.26 |

*Note:* * interventional odds ratio is significantly different from zero at the 5% level

**Table D5: The estimated effect of social influences on COVID-19 vaccine intention (from low intention to high intention) from interventional query on the learnt Bayesian network model**

| **Evidence (intervened) variable** | **Evidence variable reference level** | **Evidence variable target level** | **Interventional odds ratio** | **Lower 95% confidence interval** | **Upper 95% confidence interval** |
| --- | --- | --- | --- | --- | --- |
| proportion of community that I think will take the COVID-19 vaccine | Fewer than half | More than half | 2.44* | 2.08 | 2.85 |
| reference community** expects me to take COVID-19 vaccine | No | Yes | 1.04 | 0.89 | 1.22 |
| reference community expects me to take COVID-19 vaccine | No | Unsure | 1.02 | 0.88 | 1.19 |
| reference community considers COVID-19 a serious threat | No | Yes | 1.00 | 0.86 | 1.17 |
| reference community considers COVID-19 a serious threat | No | Unsure | 1.00 | 0.86 | 1.17 |

*Note 1:* * interventional odds ratio is significantly different from zero at the 5% level

*Note 2:* ** respondents mostly specified their immediate family and friends as their reference community

**Table D6: The estimated effect of structural enablers/barriers on COVID-19 vaccine intention (from low intention to high intention) from interventional query on the learnt Bayesian network model**

| **Evidence (intervened) variable** | **Evidence variable reference level** | **Evidence variable target level** | **Interventional odds ratio** | **Lower 95% confidence interval** | **Upper 95% confidence interval** |
| --- | --- | --- | --- | --- | --- |
| has health insurance | No | Yes | 1.02 | 0.87 | 1.20 |
| has personal care physician | No | Yes | 1.06 | 0.91 | 1.24 |
| easy to get COVID-19 vaccine once it is available | No | Yes | 1.01 | 0.86 | 1.17 |
| delayed medical care in the past year due to cost | No | Yes | 1.00 | 0.85 | 1.17 |
| delayed medical care in the past year due to work schedule | No | Yes | 1.00 | 0.85 | 1.17 |
| delayed medical care in the past year due to childcare | Yes | No | 1.17* | 1.01 | 1.37 |

*Note:* * interventional odds ratio is significantly different from zero at the 5% level

**Table D7: The estimated effect of emotions and behaviours/actions on COVID-19 vaccine intention (from low intention to high intention) from interventional query on the learnt Bayesian network model**

| **Evidence (intervened) variable** | **Evidence variable reference level** | **Evidence variable target level** | **Interventional odds ratio** | **Lower 95% confidence interval** | **Upper 95% confidence interval** |
| --- | --- | --- | --- | --- | --- |
| **Emotions** | | | | | |
| worries about catching COVID-19 | Not at all/Not much | Moderate amount | 1.18* | 1.01 | 1.38 |
| worries about catching COVID-19 | Not at all/Not much | A great deal | 1.17* | 1.00 | 1.37 |
| regret if I did not take COVID vaccine and then caught COVID-19 | No | Yes | 3.24* | 2.76 | 3.81 |
| **Behaviours** | | | | | |
| willing to take the COVID-19 vaccine in the first three months | No | Yes | 2769.89*＾ | 297.21 | 25814.17 |
| willing to take the COVID-19 vaccine in the first three months | No | Unsure | 0.71* | 0.61 | 0.82 |
| seek medical care if I have symptoms | No | Yes | 1.00 | 0.86 | 1.19 |

*Note 1: * interventional odds ratio is significantly different from zero at the 5% level*

*Note 2:＾High OR: among respondents with low vaccine intention, there is only 1 early adopter, whereas close to 75% of respondents with high vaccine intention are early adopters. This variable serves more as a proxy for vaccine.*

Tables D8-D14 present interventional ORs – the ratio of odds of **high** vaccine intention and **moderate** vaccine intention when an intervention is deployed or not. Interventional ORs are computed from the results of interventional queries that are performed on the BN model learnt from the Surgo COVID-19 Vaccine Survey data. The interventional odd ratios are also summarized graphically in Figure D2.

**Table D8: The estimated effect of beliefs and perception on COVID-19 vaccine intention (from moderate intention to high intention) from interventional query on the learnt Bayesian network model**

| **Evidence (intervened) variable** | **Evidence variable reference level** | **Evidence variable target level** | **Interventional odds ratio** | **Lower 95% confidence interval** | **Upper 95% confidence interval** |
| --- | --- | --- | --- | --- | --- |
| believes COVID-19 vaccine is free | No | Yes | 1.00 | 0.88 | 1.13 |
| believes COVID-19 vaccine is tested for the safety and effectiveness of my race | Disagree | Agree | 1.00 | 0.88 | 1.14 |
| believes COVID-19 vaccine is tested for the safety and effectiveness of my race | Disagree | Neither agree or disagree | 1.00 | 0.88 | 1.14 |
| believes people of my race are fairly treated in a health-care setting | No | Yes | 1.00 | 0.88 | 1.13 |
| trust in vaccine development process of pharmaceutical firms | Disagree | Agree | 1.61* | 1.42 | 1.83 |
| believes COVID vaccine will be unsafe | No | Yes | 0.88 | 0.77 | 1.00 |
| believes that COVID-19 testing is too rushed | No | Yes | 1.00 | 0.88 | 1.14 |
| believes in having responsibility to get vaccinated for COVID-19 to protect others | No | Yes | 7.97* | 6.87 | 9.25 |
| COVID-19 is perceived to be dangerous to my community | No | Yes | 1.21* | 1.06 | 1.37 |
| believes that a tracking chip is implanted in me through vaccine | No | Yes | 1.00 | 0.88 | 1.14 |
| has control over getting COVID | No | Yes | 1.01 | 0.89 | 1.14 |

*Note:* * interventional odds ratio is significantly different from zero at the 5% level

**Table D9: The estimated effect of outcome expectations on COVID-19 vaccine intention (from moderate intention to high intention) from interventional query on the learnt Bayesian network model**

| **Evidence (intervened) variable** | **Evidence variable reference level** | **Evidence variable target level** | **Interventional odds ratio** | **Lower 95% confidence interval** | **Upper 95% confidence interval** |
| --- | --- | --- | --- | --- | --- |
| expected chance of getting seriously ill from COVID-19 | Low | Moderate | 1.00 | 0.88 | 1.14 |
| expected chance of getting seriously ill from COVID-19 | Low | High | 1.01 | 0.89 | 1.14 |
| expected chance of getting COVID with no vaccine | Low | Moderate | 1.05 | 0.92 | 1.19 |
| expected chance of getting COVID with no vaccine | Low | High | 1.08 | 0.96 | 1.23 |
| expected chance of dying from COVID with no vaccine | Low | Moderate | 1.00 | 0.88 | 1.14 |
| expected chance of dying from COVID with no vaccine | Low | High | 1.01 | 0.89 | 1.15 |
| expected chance of getting long-term side-effects from COVID vaccine | Low | Moderate | 1.00 | 0.88 | 1.14 |
| expected chance of getting long-term side-effects from COVID vaccine | Low | High | 1.00 | 0.88 | 1.14 |

*Note:* * interventional odds ratio is significantly different from zero at the 5% level

**Table D10: The estimated effect of demographic factors on COVID-19 vaccine intention (from moderate intention to high intention) from interventional query on the learnt Bayesian network model**

| **Evidence (intervened) variable** | **Evidence variable reference level** | **Evidence variable target level** | **Interventional odds ratio** | **Lower 95% confidence interval** | **Upper 95% confidence interval** |
| --- | --- | --- | --- | --- | --- |
| gender | Man | Woman | 1.01 | 0.89 | 1.14 |
| race | White | Black | 1.00 | 0.88 | 1.13 |
| race | White | Other minorities | 1.00 | 0.88 | 1.13 |
| political affiliation | Republican | Democrat | 1.13 | 1.00 | 1.28 |
| political affiliation | Republican | Independent | 1.03 | 0.91 | 1.17 |
| education | High school or less | Some college or associates | 1.02 | 0.90 | 1.16 |
| education | High school or less | Bachelor's degree or more | 1.03 | 0.91 | 1.17 |
| age | 18-34 | 35-64 | 1.04 | 0.91 | 1.18 |
| age | 18-34 | 65 or over | 1.08 | 0.95 | 1.22 |
| is essential worker | No | Yes | 1.00 | 0.88 | 1.13 |
| urbanicity | Urban | Rural | 0.99 | 0.88 | 1.13 |
| income | Less than $30,000 | $30,000 to under $100,000 | 1.00 | 0.88 | 1.13 |
| income | Less than $30,000 | $100,000 or more | 1.00 | 0.88 | 1.13 |

*Note:* * interventional odds ratio is significantly different from zero at the 5% level

**Table D11: The estimated effect of influencers on COVID-19 vaccine intention (from moderate intention to high intention) from interventional query on the learnt Bayesian network model**

| **Evidence (intervened) variable** | **Evidence variable reference level** | **Evidence variable target level** | **Interventional odds ratio** | **Lower 95% confidence interval** | **Upper 95% confidence interval** |
| --- | --- | --- | --- | --- | --- |
| believes in COVID-19 information from Trump | Disagree | Agree | 0.96 | 0.84 | 1.08 |
| believes in COVID-19 information from Trump | Disagree | Sometimes agree or disagree | 0.96 | 0.85 | 1.09 |
| believes in COVID-19 information from public health organizations | Disagree | Agree | 1.00 | 0.88 | 1.13 |
| believes in COVID-19 information from public health organizations | Disagree | Sometimes agree or disagree | 1.00 | 0.88 | 1.13 |
| believes in COVID-19 information from my physician | Disagree | Agree | 1.00 | 0.88 | 1.13 |
| believes in COVID-19 information from my physician | Disagree | Sometimes agree or disagree | 1.00 | 0.88 | 1.13 |
| believes in COVID-19 information from my local community | Disagree | Agree | 1.00 | 0.88 | 1.13 |
| believes in COVID-19 information from my local community | Disagree | Sometimes agree or disagree | 1.00 | 0.88 | 1.13 |
| believes in COVID-19 information from my social media contacts | Disagree | Agree | 1.01 | 0.89 | 1.14 |
| believes in COVID-19 information from my social media contacts | Disagree | Sometimes agree or disagree | 0.99 | 0.88 | 1.13 |
| gets information on COVID-19 from left-wing media | No | Yes | 1.00 | 0.88 | 1.13 |
| gets information on COVID-19 from Fox News | No | Yes | 1.00 | 0.88 | 1.13 |
| gets information on COVID-19 from social media | No | Yes | 1.00 | 0.88 | 1.14 |
| gets information on COVID-19 from scientists | No | Yes | 1.04 | 0.92 | 1.18 |

*Note:* * interventional odds ratio is significantly different from zero at the 5% level

**Table D12: The estimated effect of social influences on COVID-19 vaccine intention (from moderate intention to high intention) from interventional query on the learnt Bayesian network model**

| **Evidence (intervened) variable** | **Evidence variable reference level** | **Evidence variable target level** | **Interventional odds ratio** | **Lower 95% confidence interval** | **Upper 95% confidence interval** |
| --- | --- | --- | --- | --- | --- |
| proportion of community that I think will take the COVID-19 vaccine | Fewer than half | More than half | 1.66* | 1.46 | 1.89 |
| reference community** expects me to take COVID-19 vaccine | No | Yes | 1.02 | 0.90 | 1.16 |
| reference community expects me to take COVID-19 vaccine | No | Not sure | 1.01 | 0.89 | 1.14 |
| reference community considers COVID-19 a serious threat | No | Yes | 1.00 | 0.88 | 1.13 |
| reference community considers COVID-19 a serious threat | No | Not sure | 1.00 | 0.88 | 1.13 |

*Note 1:* * interventional odds ratio is significantly different from zero at the 5% level

*Note 2:* ** respondents mostly specified their immediate family and friends as their reference community

**Table D13: The estimated effect of structural enablers/barriers on COVID-19 vaccine intention (from moderate intention to high intention) from interventional query on the learnt Bayesian network model**

| **Evidence (intervened) variable** | **Evidence variable reference level** | **Evidence variable target level** | **Interventional odds ratio** | **Lower 95% confidence interval** | **Upper 95% confidence interval** |
| --- | --- | --- | --- | --- | --- |
| has health insurance | 0 | 1 | 1.01 | 0.89 | 1.15 |
| has personal care physician | 0 | 1 | 1.04 | 0.91 | 1.17 |
| easy to get COVID-19 vaccine once it is available | 0 | 1 | 1.00 | 0.88 | 1.14 |
| delayed medical care in the past year due to cost | 0 | 1 | 1.00 | 0.88 | 1.13 |
| delayed medical care in the past due to work schedule | 0 | 1 | 1.00 | 0.88 | 1.13 |
| delayed medical care in the past due to childcare | 0 | 1 | 0.91 | 0.80 | 1.03 |

*Note:* * interventional odds ratio is significantly different from zero at the 5% level

**Table D14: The estimated effect of emotions and behaviours/actions on COVID-19 vaccine intention (from moderate intention to high intention) from interventional query on the learnt Bayesian network model**

| **Evidence (intervened) variable** | **Evidence variable reference level** | **Evidence variable target level** | **Interventional odds ratio** | **Lower 95% confidence interval** | **Upper 95% confidence interval** |
| --- | --- | --- | --- | --- | --- |
| worries about catching COVID-19 | Not at all or not much | Moderate | 1.09 | 0.96 | 1.23 |
| worries about catching COVID-19 | Not at all or not much | A great deal | 1.08 | 0.96 | 1.23 |
| willing to take the COVID-19 vaccine in the first three months | No | Yes | 19.31* | 16.44 | 22.67 |
| willing to take the COVID-19 vaccine in the first three months | No | Not sure | 1.90* | 1.65 | 2.19 |
| regret if I did not take COVID-19 vaccine and then caught COVID-19 | No | Yes | 1.74* | 1.53 | 1.97 |
| seek check-up if I have symptoms in general | No | Yes | 1.01 | 0.89 | 1.14 |

*Note:* * interventional odds ratio is significantly different from zero at the 5% level


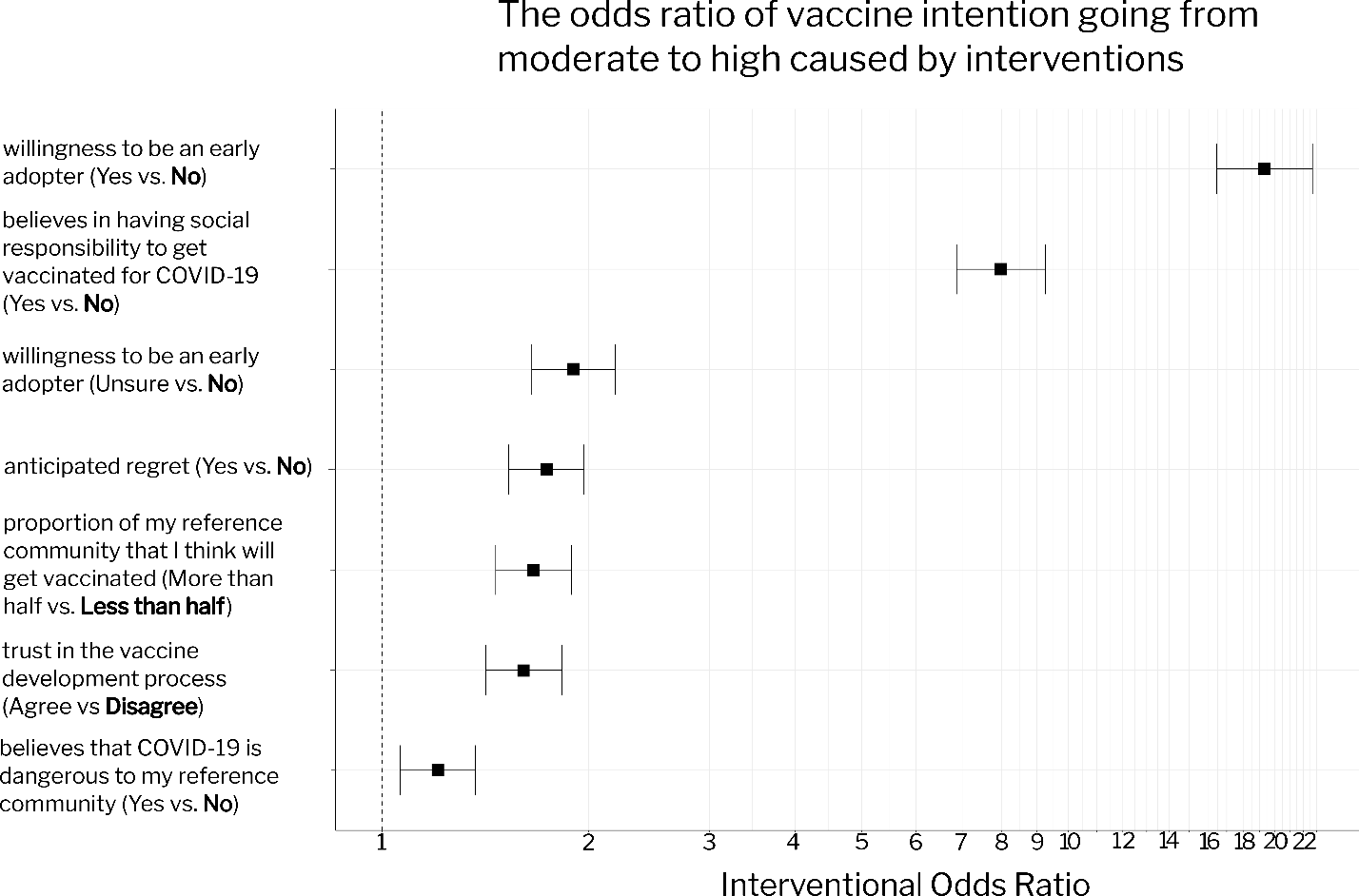


**Figure D2:** The interventional odds ratio (OR) of factors that have statistically significant estimated causal effects (at the 5% level) on vaccine. The bold text in the y-axis labels indicates the reference level of a given variable, and the error bars are the 95% confidence interval of the OR estimate. The results suggest that making people believe that it is their social responsibility to get vaccinated to protect others, being an early adopter, triggering anticipated regret of not taking the vaccine, making people believe that the majority of their community is taking the vaccine, and making people trust the vaccine development have the largest effect in increasing vaccine intention from moderate to high.

# **Appendix E: Interventional Queries on Causes of Cause**

Tables E1-E16 present the interventional ORs for outcome variables *other than vaccine intention*. The outcome variables in this set of analyses are the direct/indirect causes of vaccine intention (rather than vaccine intention). The interventional ORs are computed from the results of interventional queries that are performed on the BN model trained from the Surgo COVID-19 Vaccine Survey data. These results help us establish various causal pathways of vaccine intention.

Here, we present the detailed results on the determinants of three important direct causes of vaccine intention – *social responsibility, belief that vaccines will be unsafe,* and *anticipated regret.*

We found that *social responsibility* (Table E6) is driven by the perceived risk of COVID-19 to self and others: *the expected chance of getting COVID-19 without vaccines* (OR=1.16, 95% CI=1.04 – 1.30), *the level of worry about getting COVID* (OR=1.15, 95% CI=1.03 – 1.29), and *the belief that COVID is dangerous to one's community* (OR=1.18, 95% CI=1.05 – 1.31). Since individual effects of these factors are small, intervening on them simultaneously might be required to promote *social responsibility*.

In a similar manner, we found that *the belief that vaccines will be unsafe* (Table E1) could be influenced by interventions that promote trust in the vaccine development process (OR= 0.366, 95% CI=0.318 – 0.421), show people that the majority of their community are taking the COVID-19 vaccine (OR=0.294, 95% CI=0.251 – 0.345), promote social responsibility (OR=0.250, 95% CI=0.211 – 0.295), and show people that they might feel regret if they did not take the vaccine (OR=0.477, 95% CI=0.415 – 0.550).

*Anticipated regret*, the third direct causal factor of vaccine intention revealed by the analysis, is driven by variables that fall into the following classes (Table E2): perceived risk of COVID-19 to self and others, social considerations*,* and vaccine safety concerns. We found that intervening on each of these variables individually had a small effect on anticipated regret (OR<=1.45). This suggests that anticipated regret will require a more concerted effort to nudge.

**Table E1: The interventional odds ratio for “believes COVID vaccine will be unsafe” (reference level: No; target level: Yes) that are statistically significant at the 5% level**

| **Evidence (intervened) variable** | **Evidence variable reference level** | **Evidence variable target level** | **Interventional odds ratio** | **Lower 95% confidence interval** | **Upper 95% confidence interval** |
| --- | --- | --- | --- | --- | --- |
| trust in vaccine development process of pharmaceutical firms | Disagree | Agree | 0.366 | 0.318 | 0.421 |
| believes in having responsibility to get vaccinated for COVID-19 to protect others | No | Yes | 0.250 | 0.211 | 0.295 |
| proportion of community that I think will take the COVID-19 vaccine | Fewer than half | More than half | 0.294 | 0.251 | 0.345 |
| willing to take the COVID-19 vaccine in the first three months | No | Yes | 0.175 | 0.144 | 0.211 |
| willing to take the COVID-19 vaccine in the first three months | No | Unsure | 1.369 | 1.210 | 1.548 |
| COVID-19 is perceived to be dangerous to my community | No | Yes | 0.732 | 0.629 | 0.851 |
| regret if I did not take COVID -19 vaccine and then caught COVID-19 | No | Yes | 0.477 | 0.415 | 0.550 |

**Table E2: The interventional odds ratio for “regret if I did not take vaccines and then caught COVID-19” (reference level: No; target level: Yes) that are statistically significant at the 5% level**

| **Evidence (intervened) variable** | **Evidence variable reference level** | **Evidence variable target level** | **Interventional odds ratio** | **Lower 95% confidence interval** | **Upper 95% confidence interval** |
| --- | --- | --- | --- | --- | --- |
| **Perceived risk of COVID-19 to self and others** | | | | | |
| expected chance of getting COVID with no vaccine | Low | High | 1.12 | 1.00 | 1.25 |
| worries about catching COVID-19 | Not at all or not much | Moderate | 1.17 | 1.05 | 1.31 |
| believes COVID-19 is dangerous for my community | No | Yes | 1.24 | 1.11 | 1.39 |
| **Vaccine safety concerns** | | | | | |
| trust in vaccine development process of pharmaceutical firms | Disagree | Agree | 1.14 | 1.02 | 1.27 |
| **Social considerations** | | | | | |
| believes in having responsibility to get vaccinated for COVID-19 to protect others | No | Yes | 1.45 | 1.30 | 1.62 |
| reference community* expects me to take COVID-19 vaccine | No | Yes | 1.16 | 1.03 | 1.29 |
| proportion of community that I think will take the COVID-19 vaccine | Fewer than half | More than half | 1.29 | 1.15 | 1.44 |
| **Others** | | | | | |
| willing to take the COVID-19 vaccine in the first three months | No | Yes | 1.14 | 1.02 | 1.27 |
| political affiliation | Republican | Democrat | 1.19 | 1.06 | 1.33 |

*Note 1:* * respondents mostly specified their immediate family and friends as their reference community

**Table E3: The interventional odds ratio for “willing to take vaccine in the first 3 months” (reference level: No; target level: Yes) that are statistically significant at the 5% level**

| **Evidence (intervened) variable** | **Evidence variable reference level** | **Evidence variable target level** | **Interventional odds ratio** | **Lower 95% confidence interval** | **Upper 95% confidence interval** |
| --- | --- | --- | --- | --- | --- |
| proportion of community that I think will take the COVID-19 vaccine | Fewer than half | More than half | 2.44 | 2.11 | 2.83 |
| believes in having responsibility to get vaccinated for COVID-19 to protect others | No | Yes | 16.56 | 13.79 | 19.88 |
| believes COVID-19 is dangerous for my community | No | Yes | 1.29 | 1.12 | 1.49 |

**Table E4: The interventional odds ratios for “trust in vaccine development process of pharmaceutical firms” (reference level: Disagree; target level: Agree) that are statistically significant at the 5% level**

| **Evidence (intervened) variable** | **Evidence variable reference level** | **Evidence variable target level** | **Interventional odds ratio** | **Lower 95% confidence interval** | **Upper 95% confidence interval** |
| --- | --- | --- | --- | --- | --- |
| believes in having responsibility to get vaccinated for COVID-19 to protect others | No | Yes | 6.35 | 5.38 | 7.49 |
| proportion of community that I think will take the COVID-19 vaccine | Fewer than half | More than half | 2.68 | 2.33 | 3.08 |
| has primary care physician | No | Yes | 1.50 | 1.31 | 1.71 |
| delayed medical care in the past year due to childcare | No | Yes | 0.83 | 0.73 | 0.95 |
| believes COVID-19 is dangerous for my community | No | Yes | 1.29 | 1.12 | 1.47 |
| age | 18-34 | 65 or over | 1.23 | 1.07 | 1.42 |

**Table E5: The interventional odds ratios for “proportion of community that I think will take the COVID-19 vaccine” (reference level: Fewer than half; target level: More than half) that are statistically significant at the 5% level**

| **Evidence (intervened) variable** | **Evidence variable reference level** | **Evidence variable target level** | **Interventional odds ratio** | **Lower 95% confidence interval** | **Upper 95% confidence interval** |
| --- | --- | --- | --- | --- | --- |
| believes in having responsibility to get vaccinated for COVID-19 to protect others | No | Yes | 8.43 | 7.38 | 9.62 |
| delayed medical care in the past year due to childcare | No | Yes | 0.48 | 0.43 | 0.54 |
| believes COVID-19 is dangerous for my community | No | Yes | 2.31 | 2.06 | 2.60 |
| political affiliation | Republican | Democrat | 1.35 | 1.21 | 1.52 |
| age | 18-34 | 35-64 | 1.15 | 1.02 | 1.29 |
| age | 18-34 | 65 or over | 1.27 | 1.13 | 1.42 |

**Table E6: The interventional odds ratios for “believes in having responsibility to get vaccinated for COVID-19 to protect others” (reference level: No; target level: Yes) that are statistically significant at the 5% level**

| **Evidence (intervened) variable** | **Evidence variable reference level** | **Evidence variable target level** | **Interventional odds ratio** | **Lower 95% confidence interval** | **Upper 95% confidence interval** |
| --- | --- | --- | --- | --- | --- |
| **Demographics** | | | | | |
| political affiliation | Republican | Democrat | 1.18 | 1.06 | 1.32 |
| **Perceived risk of COVID-19 to self and others** | | | | | |
| expected chance of getting COVID-19 with no vaccine | Low | High | 1.16 | 1.04 | 1.30 |
| worries about catching COVID-19 | Not at all | Moderate Amount | 1.15 | 1.03 | 1.29 |
| worries about catching COVID-19 | Not at all | A great deal | 1.16 | 1.04 | 1.30 |
| believes COVID-19 is dangerous for my community | No | Yes | 1.18 | 1.05 | 1.31 |

**Table E7: The interventional odds ratios for “expected chance of getting COVID-19 with no vaccine” (reference level: Low; target level: High) that are statistically significant at the 5% level**

| **Evidence (intervened) variable** | **Evidence variable reference level** | **Evidence variable target level** | **Interventional odds ratio** | **Lower 95% confidence interval** | **Upper 95% confidence interval** |
| --- | --- | --- | --- | --- | --- |
| believes COVID-19 is dangerous for my community | No | Yes | 6.29 | 5.35 | 7.41 |
| worries about catching COVID-19 | Not at all | Moderate Amount | 7.19 | 6.03 | 8.58 |
| worries about catching COVID-19 | Not at all | A great deal | 20.18 | 16.91 | 24.07 |
| gets information on COVID-19 from scientists | No | Yes | 1.16 | 1.00 | 1.34 |
| race | White | Black | 1.53 | 1.31 | 1.77 |
| race | White | Other minorities | 1.17 | 1.01 | 1.35 |
| political affiliation | Republican | Democrat | 2.89 | 2.48 | 3.37 |
| political affiliation | Republican | Independent | 1.42 | 1.23 | 1.65 |
| age | 18-34 | 35-64 | 1.20 | 1.03 | 1.39 |
| age | 18-34 | 65 or over | 1.33 | 1.15 | 1.55 |

**Table E8: The interventional odds ratios for “expected chance of getting COVID-19 with no vaccine” (reference level: Low; target level: Moderate) that are statistically significant at the 5% level**

| **Evidence (intervened) variable** | **Evidence variable reference level** | **Evidence variable target level** | **Interventional odds ratio** | **Lower 95% confidence interval** | **Upper 95% confidence interval** |
| --- | --- | --- | --- | --- | --- |
| believes COVID-19 is dangerous for my community | No | Yes | 2.46 | 2.14 | 2.83 |
| worries about catching COVID-19 | Not at all | Moderate Amount | 3.40 | 2.97 | 3.90 |
| worries about catching COVID-19 | Not at all | A great deal | 1.93 | 1.64 | 2.26 |
| race | White | Black | 1.24 | 1.07 | 1.42 |
| political affiliation | Republican | Democrat | 1.69 | 1.46 | 1.94 |
| political affiliation | Republican | Independent | 1.17 | 1.03 | 1.33 |
| age | 18-34 | 65 or over | 1.16 | 1.01 | 1.34 |

**Table E9: The interventional odds ratios for “expected chance of getting COVID-19 with no vaccine” (reference level: Moderate; target level: High) that are statistically significant at the 5% level**

| **Evidence (intervened) variable** | **Evidence variable reference level** | **Evidence variable target level** | **Interventional odds ratio** | **Lower 95% confidence interval** | **Upper 95% confidence interval** |
| --- | --- | --- | --- | --- | --- |
| believes COVID-19 is dangerous for my community | No | Yes | 2.56 | 2.21 | 2.97 |
| worries about catching COVID-19 | Not at all | Moderate Amount | 2.11 | 1.80 | 2.48 |
| worries about catching COVID-19 | Not at all | A great deal | 10.48 | 8.90 | 12.34 |
| race | White | Black | 1.23 | 1.08 | 1.41 |
| political affiliation | Republican | Democrat | 1.72 | 1.50 | 1.96 |
| political affiliation | Republican | Independent | 1.22 | 1.06 | 1.40 |

**Table E10: The interventional odds ratios for “worries about catching COVID-19” (reference level: Not at all; target level: A great deal) that are statistically significant at the 5% level**

| **Evidence (intervened) variable** | **Evidence variable reference level** | **Evidence variable target level** | **Interventional odds ratio** | **Lower 95% confidence interval** | **Upper 95% confidence interval** |
| --- | --- | --- | --- | --- | --- |
| gets COVID-19 information from scientists | No | Yes | 1.30 | 1.11 | 1.52 |
| believes COVID-19 is dangerous for my community | No | Yes | 14.19 | 11.41 | 17.66 |
| race | White | Black | 2.16 | 1.85 | 2.54 |
| race | White | Other minorities | 1.32 | 1.12 | 1.54 |
| political affiliation | Republican | Democrat | 7.14 | 5.99 | 8.50 |
| political affiliation | Republican | Independent | 1.87 | 1.57 | 2.23 |
| age | 18-34 | 35-64 | 1.25 | 1.07 | 1.47 |
| age | 18-34 | 65 or over | 1.43 | 1.22 | 1.68 |

**Table E11: The interventional odds ratios for “worries about catching COVID-19” (reference level: Not at all; target level: Moderate amount) that are statistically significant at the 5% level**

| **Evidence (intervened) variable** | **Evidence variable reference level** | **Evidence variable target level** | **Interventional odds ratio** | **Lower 95% confidence interval** | **Upper 95% confidence interval** |
| --- | --- | --- | --- | --- | --- |
| gets COVID-19 information from scientists | No | Yes | 1.21 | 1.07 | 1.37 |
| believes COVID-19 is dangerous for my community | No | Yes | 4.55 | 3.99 | 5.18 |
| race | White | Black | 1.77 | 1.55 | 2.01 |
| race | White | Other minorities | 1.22 | 1.07 | 1.38 |
| political affiliation | Republican | Democrat | 4.12 | 3.61 | 4.72 |
| political affiliation | Republican | Independent | 1.44 | 1.28 | 1.63 |
| age | 18-34 | 35-64 | 1.17 | 1.03 | 1.32 |
| age | 18-34 | 65 or over | 1.29 | 1.14 | 1.46 |

**Table E12: The interventional odds ratios for “worries about catching COVID-19” (reference level: Moderate amount; target level: A great deal) that are statistically significant at the 5% level**

| **Evidence (intervened) variable** | **Evidence variable reference level** | **Evidence variable target level** | **Interventional odds ratio** | **Lower 95% confidence interval** | **Upper 95% confidence interval** |
| --- | --- | --- | --- | --- | --- |
| believes COVID-19 is dangerous for my community | No | Yes | 3.12 | 2.51 | 3.88 |
| race | White | Black | 1.23 | 1.06 | 1.42 |
| political affiliation | Republican | Democrat | 1.73 | 1.47 | 2.04 |
| political affiliation | Republican | Independent | 1.30 | 1.09 | 1.56 |

**Table E13: The interventional odds ratios for “believes COVID-19 is dangerous for my community” (reference level: No; target level: Yes) that are statistically significant at the 5% level**

| **Evidence (intervened) variable** | **Evidence variable reference level** | **Evidence variable target level** | **Interventional odds ratio** | **Lower 95% confidence interval** | **Upper 95% confidence interval** |
| --- | --- | --- | --- | --- | --- |
| gets COVID-19 information from scientists | No | Yes | 1.20 | 1.06 | 1.35 |
| race | White | Black | 1.73 | 1.53 | 1.95 |
| race | White | Other minorities | 1.21 | 1.08 | 1.36 |
| political affiliation | Republican | Democrat | 3.93 | 3.46 | 4.46 |
| political affiliation | Republican | Independent | 1.63 | 1.46 | 1.83 |
| age | 18-34 | 35-64 | 1.57 | 1.40 | 1.76 |
| age | 18-34 | 65 or over | 2.13 | 1.89 | 2.41 |

**Table E14: The interventional odds ratios for “easy to get COVID-19 vaccine once it is available” (reference level: No; target level: Yes) that are statistically significant at the 5% level**

| **Evidence (intervened) variable** | **Evidence variable reference level** | **Evidence variable target level** | **Interventional odds ratio** | **Lower 95% confidence interval** | **Upper 95% confidence interval** |
| --- | --- | --- | --- | --- | --- |
| has health insurance | No | Yes | 2.63 | 2.34 | 2.95 |
| trust in vaccine development process of pharmaceutical firms | No | Yes | 1.64 | 1.46 | 1.83 |
| believes in having responsibility to get vaccinated for COVID-19 to protect others | No | Yes | 1.15 | 1.03 | 1.28 |
| education | High school or less | Bachelor's degree or more | 1.16 | 1.03 | 1.29 |
| age | 18-34 | 35-64 | 1.13 | 1.01 | 1.26 |
| age | 18-34 | 65 or over | 1.35 | 1.21 | 1.52 |
| seek medical check-up if I have symptoms | No | Yes | 1.60 | 1.43 | 1.79 |

**Table E15: The interventional odds ratios for “seek medical check-up if I have symptoms” (reference level: No; target level: Yes) that are statistically significant at the 5% level**

| **Evidence (intervened) variable** | **Evidence variable reference level** | **Evidence variable target level** | **Interventional odds ratio** | **Lower 95% confidence interval** | **Upper 95% confidence interval** |
| --- | --- | --- | --- | --- | --- |
| has health insurance | No | Yes | 3.82 | 3.30 | 4.42 |
| delayed medical care in the past year due to work schedule | No | Yes | 0.41 | 0.35 | 0.48 |
| delayed medical care in the past year due to childcare | No | Yes | 0.74 | 0.63 | 0.85 |
| education | High school or less | Bachelor's degree or more | 1.28 | 1.09 | 1.50 |
| age | 18-34 | 35-64 | 1.71 | 1.48 | 1.98 |
| age | 18-34 | 65 or over | 8.01 | 6.35 | 10.09 |

**Table E16: The interventional odds ratios for “political affiliation” (reference level: Republican; target level: Democrat). Only evidence variables that are direct or indirect causes of political affiliation from the BN are included**

| **Evidence (intervened) variable** | **Evidence variable reference level** | **Evidence variable target level** | **Interventional odds ratio** | **Lower 95% confidence interval** | **Upper 95% confidence interval** |
| --- | --- | --- | --- | --- | --- |
| gets information on COVID-19 from scientists | No | Yes | 2.33 | 2.02 | 2.69 |
| race | White | Black | 23.59 | 18.84 | 29.53 |
| race | White | Other minorities | 2.58 | 2.24 | 2.98 |
| education | High school or less | Some college or associates | 1.12 | 0.98 | 1.29 |
| education | High school or less | Bachelor's degree or more | 1.24 | 1.08 | 1.43 |
| age | 18-34 | 35-64 | 0.99 | 0.86 | 1.13 |
| age | 18-34 | 65 or over | 0.98 | 0.86 | 1.13 |

# **Appendix F: Weighted Least Square models with variables related to political affiliation**

Using simple Weighted Least Squares (WLS) models, we estimated the association between “Republican” – a binary variable that takes on “Yes” for respondents who reported to be Republicans, and “No” for respondents who reported to be Democrats or Independents – and vaccine intention.

**Table F1: Estimated association between Republican and vaccine intention**

| **Independent variable \Dependent variable** | | **Republican** |
| --- | --- | --- |
| Vaccine intention: High (vs Low) | Regression coefficients (log-odds) | -0.856*** |
|  | p-value | 0.000 |
|  | 95% CI | (-1.128, -0.584) |
| Vaccine intention: Moderate (vs Low) | Regression coefficients (log-odds) | -0.406** |
|  | p-value | 0.0160 |
|  | 95% CI | (-0.734, -0.078) |
| Constant | Regression coefficients (log-odds) | -0.291*** |
|  | p-value | 0.006 |
|  | 95% CI | (-0.496, -0.086) |

Note: *p<0.1; **p<0.05; ***p<0.01

# **Appendix G: Using the BN to infer the impact of political affiliation**

We highlighted in a use case a key advantage of BN: the same model can be used to estimate impacts of alternative cause-effect pairs. This is because there is no need to pre-classify variables as inputs (causes) or outputs (effects) in a BN, and multiple causal cascades can be compactly represented. Here, we used our learnt BN from the Surgo COVID-19 Vaccine Survey data to estimate the influence of political affiliations on intermediary causes of vaccine intention. We did this by considering in the DAG only the pathways connecting political affiliation to vaccine intention, while ignoring any variables that are upstream of political affiliation.

Figure G1 shows the causal pathway from political affiliation to vaccine intention. Political affiliation, which is a remote driver of vaccine intention, has three direct effects – *social responsibility*, *the level of worry about getting COVID*, and *the belief that COVID is dangerous to one's community.* Note that the pathways from political affiliation to vaccine intention includes most of the significant causal factors of vaccine intention. With the exception of *delayed medical care due to childcare*, most of the significant causal factors of vaccine intention are conditionally dependent on political affiliation, but this need not be the case in general.


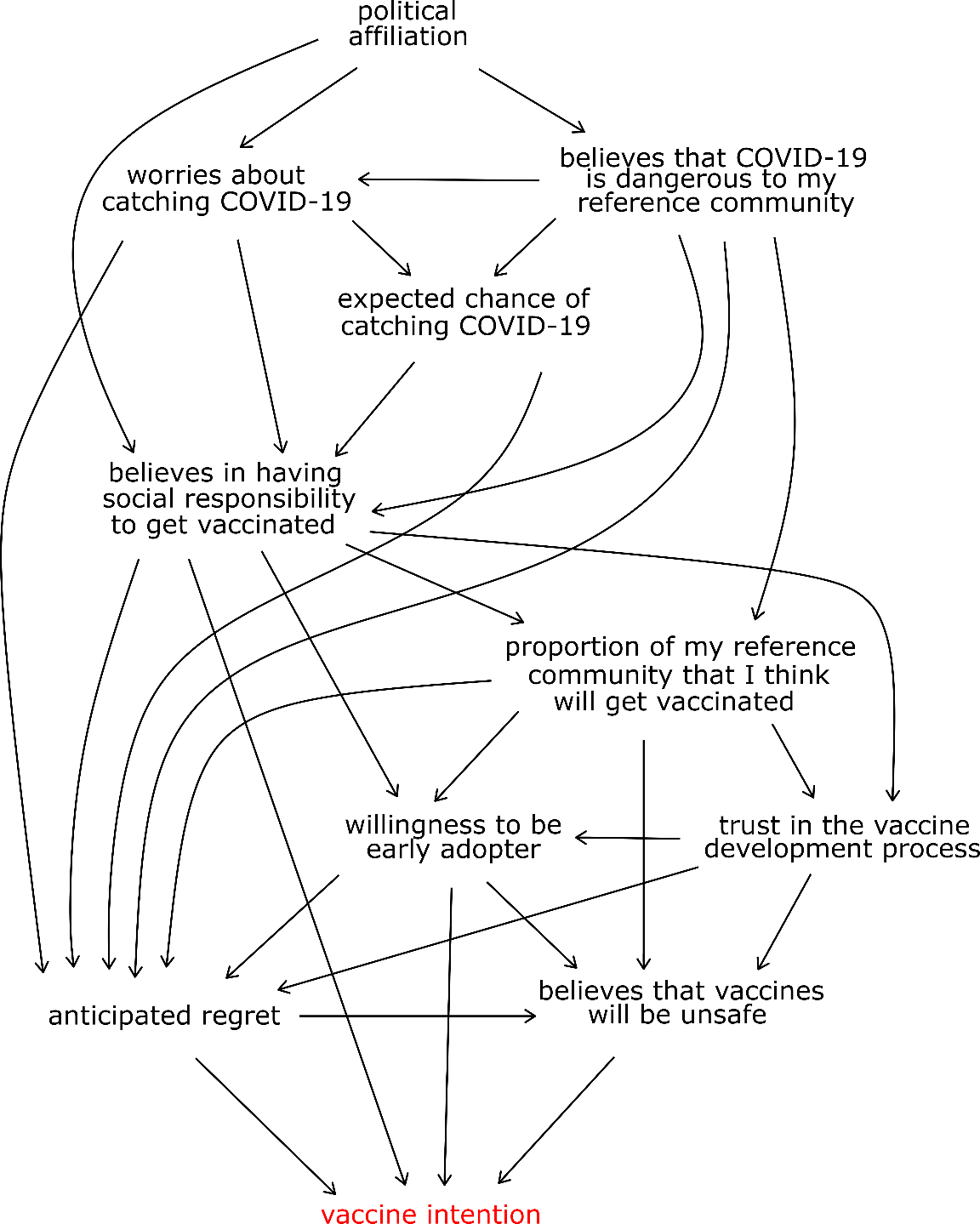


**Figure G1:** The direct and indirect causes of political affiliation. The estimated causal effect of political affiliation on vaccine intention is statistically significant but not substantial: it is mostly mediated by *worries about catching COVID-19* and *believe that COVID-19 is dangerous to my community.*

Although *social responsibility* is a direct and strong causal effect on vaccine intention, it is only weakly influenced by *political affiliation* (OR=1.18, 95% CI=1.06 – 1.32). Instead, *political affiliation* has a larger effect on *the level of worry about getting COVID-19* (OR=7.14, 95% CI=5.99 – 8.50) and *the belief that COVID-19 is dangerous to one's community* (OR=3.93, 95% CI=3.46 – 4.46). These findings, which suggest that Republicans have lower perceptions of COVID-19 risk than Democrats, are corroborated by many US studies on vaccine hesitancy. Although it seems reasonable to believe that Republicans are less likely to get vaccinated because they have lower perceived risk of COVID-19, we found that this causal relationship is weak. In fact, *the level of worry about catching COVID-19* (OR=1.17, 95% CI=1.00 – 1.37) and *the belief that COVID-19 is dangerous to one’s community* (OR=1.42, 95% CI=1.21 – 1.66) are only remote drivers that weakly influence vaccine intention. Based on these results, it is not surprising to find that the cascading effect of *political affiliation* on vaccine intention is significant but small (OR=1.26, 95% CI=1.08 – 1.48).

The result that people’s political affiliations have only a weak causal effect on their vaccine intention seems at odds with observational studies that suggest that self-identified Republicans generally have a lower vaccine intention than individuals who identified as Democrats. Indeed, using weighted logistic regression (Appendix F), we found that on average, respondents with high vaccine intention are less likely to identify themselves as Republicans compared to respondents with low vaccine intention (log-odds =-0.856, 95% CI=-1.128 – -0.584).

This apparent discrepancy highlights the difference between a causal interpretation and a predictive (correlative) one. One explanation is the existence of confounders that are explicitly modelled in the BN and not in a correlative analysis. By definition, these confounders are common causes to political affiliation and vaccine intention, whose causal contribution to vaccine intention may be misattributed to political affiliation due to their co-occurrence with specific political affiliations by methods such as regression. Admittedly not all confounders are captured in our data; however, *age*, *race*, *education* and *getting COVID-19 information from scientists* were suggested as observed confounders by the positions of these variables in the learnt DAG (Main text, Figure 2). That is, these variables are all causal to both vaccine intention and political affiliation, even if their individual causal effect may not be statistically significant on vaccine intention. Of these, *race* and *getting COVID-19 information from scientists* – a proxy for a general trust in science – are significant causal factors (at the 5% level) of political affiliation (Table E16). Respondents who get information from scientists are more likely to self-identify as Democrats (OR=2.33, 95% CI=2.02 – 2.69), as are Black respondents (OR=23.59, 95% CI=2.02 – 2.69) and other minorities (OR=2.58, 95% CI=2.24 – 2.98) compared to White respondents in our sample. In addition, respondents who have at least a Bachelor’s degree are slightly more likely to self-identify as Democrats compared to respondents with at most high school education (OR=1.24, 95% CI=1.08 – 1.43). Although individually, none of the above factors that are significantly causal to political affiliation is also significantly causal to vaccine intention, they could still confound the relationship between political affiliation and vaccine intention in the aggregate (i.e., if these factors were to change simultaneously). Overall, the confounders would lead to an overestimation of the effect of political affiliation in non-causal analyses.

That said, descriptive statistics on observational data are still valuable if they are used as a first step of diagnosing vaccine hesitancy, or for predicting vaccination rates in different geographic areas. However, to inform the design of effective interventions, we need more rigorous approaches such as causal discovery and BN to establish causal relationships between potential drivers and vaccine intention.

# **Appendix H: Survey respondent characteristics**

**Table H1: Demographic characteristics of the participants in the Surgo COVID-19 Vaccine Survey**

| **Demographic characteristics** | **Levels of measurement** | **Number of participants in the survey (percentage)** |
| --- | --- | --- |
| Age | 18 to 29 | 470 (17.1%) |
|  | 30 to 44 | 801 (29.2%) |
|  | 45 to 69 | 574 (20.9%) |
|  | 60 or older | 902 (32.8%) |
| Gender | Male | 1357 (49.4%) |
|  | Female | 1390 (50.6%) |
|  | Other | 0 (0%) |
| Race / Ethnicity | White, non-Hispanic | 1627 (59.2%) |
|  | Black, non-Hispanic | 393 (14.3%) |
|  | Asian, non-Hispanic | 61 (2.2%) |
|  | Two or more races, non-Hispanic | 104 (3.8%) |
|  | Other, non-Hispanic | 42 (1.5%) |
|  | Hispanic | 520 (18.9%) |
| Sample Size | | 2747 |

# **Appendix I: Estimated BN Performance**

Due to the complexity of the structural learning task, there is a limited number of variables (and limited number of levels for each variable) that can be included in the BN so that it can be learnt within a reasonable amount of time. In addition, given the sample size of our vaccine survey datasets, there is a trade-off between the number of included variables and model performance.

To determine the number of variables to include in the BN, we generated multiple sets of synthetic samples with similar characteristics to our vaccine survey datasets (e.g., number of observations, and maximum number of levels associated with the variables). Each set of synthetic samples has a different number of variables (e.g., 40, 45 and 50). For each synthetic sample, we performed structural learning and then used metrics (e.g., precision, recall, and f1-score) to determine the extent to which the search algorithm has learnt the ground-truth model – the known process from which the synthetic sample was generated. After computing the aggregated performance metric for each set of synthetic samples, we determine the number of variables that could be included in our BN with acceptable model performance. Based on this analysis, we decided to include 45 variables in the BN. Table I1 presents the estimated model performance of the BN with 45 variables, 2477 observations, and at most 3 levels of measurement.

**Table I1: Model performance estimated from synthetic data**

The “skeleton” metrics measure the extent to which undirected edges in the ground-truth model are correctly estimated by the search algorithm (a variation of the Markov Chain Monte Carlo method); the “DAG” metrics take into account the estimated directions of the edges, and the “V-structure” metrics measure the extent to which V-structures (a triple where two variables is the causal parent of the third) in the ground-truth model are correctly estimated by the algorithm.

| **Estimated performance metric** | **Value** |
| --- | --- |
| Skeleton precision | 0.93 |
| Skeleton recall | 0.91 |
| Skeleton f1-score | 0.92 |
| DAG precision | 0.83 |
| DAG recall | 0.81 |
| DAG f1-score | 0.82 |
| V-structure precision | 0.92 |
| V-structure recall | 0.79 |
| V-structure f1-score | 0.82 |

# **Appendix J: Regression Analyses for Selecting Features for the Bayesian Network (BN)**

Tables J1-J5 below present results from the linear regression analyses on the determinants of vaccine intention. The purpose of this analysis is to identify factors that are highly correlated to vaccine intention. The identified factors are potential variables of interest for our subsequent Bayesian network models.

**Table J1: The estimated regression coefficients of behaviours/actions**

| **variables** | **regression coefficients** | **p-value** | **lower 95% confidence interval** | **upper 95% confidence interval** |
| --- | --- | --- | --- | --- |
| flu vaccine: Never take flu vaccine (vs. Did not take flu vaccine last year) | -0.339*** | 0.00175 | -0.625 | -0.052 |
| flu vaccine: Took flu vaccine (vs. Did not take flu vaccine last year) | 0.292*** | 0.00154 | 0.070 | 0.513 |
| willing to take the COVID-19 vaccine in the first three months: Unsure (vs. No) | -0.455*** | 0.000 | -0.740 | -0.171 |
| willing to take the COVID-19 vaccine in the first three months: Yes (vs. No) | 1.770*** | 0.000 | 1.450 | 2.090 |
| health-seeking behaviour | -0.528* | 0.0971 | -1.295 | 0.239 |
| seek check-up if I have symptoms in general | 0.140 | 0.593 | -0.532 | 0.812 |
| **Observations R^2^**  **Adjusted R^2^**  **Residual Std. Error**  **F-Statistic** | 2454 0.793 0.785 1.730 (df=2362)  99.33*** (df=91; 2362) |  |  |  |

*Note:* *p<0.1; **p<0.05; ***p<0.01

**Table J2: The estimated regression coefficients of beliefs and perceptions**

| **variables** | **regression coefficients** | **p-value** | **lower 95% confidence interval** | **upper 95% confidence interval** |
| --- | --- | --- | --- | --- |
| believes in chip implantation through vaccine: Yes (vs. No) | -0.280** | 0.0437 | -0.602 | 0.042 |
| believes natural immunity is stronger than vaccine immunity: I really have no idea (vs. No) | -0.092 | 0.482 | -0.415 | 0.232 |
| believes natural immunity is stronger than vaccine immunity: Yes (vs. No) | 0.040 | 0.709 | -0.232 | 0.312 |
| believes in having responsibility to get vaccinated for COVID-19 to protect others: Yes (vs. No) | 1.218*** | 0.000 | 0.915 | 1.521 |
| believes vaccines do more harm than good: Yes (vs. No) | -0.054 | 0.648 | -0.367 | 0.258 |
| believes vaccine will not prevent COVID-19: Yes (vs. No) | -0.276*** | 0.00418 | -0.536 | -0.016 |
| believes that COVID-19 testing is too rushed: Yes (vs. No) | -0.683*** | 0.000 | -0.944 | -0.421 |
| believes COVID vaccine will be unsafe: Yes (vs. No) | -0.699*** | 0.000 | -1.054 | -0.345 |
| COVID-19 is perceived to be dangerous to my community: Yes (vs. No) | -0.237** | 0.0133 | -0.478 | 0.004 |
| less likely to get COVID vaccine since ingredients may be incompatible with beliefs: Yes (vs. No) | -0.049 | 0.779 | -0.493 | 0.396 |
| perceived cost of COVID: Not free (vs. Free) | 0.053 | 0.514 | -0.136 | 0.241 |
| believes childhood vaccine is beneficial: I really have no idea (vs. No) | -0.280 | 0.170 | -0.811 | 0.250 |
| believes childhood vaccine is beneficial: Yes (vs. No) | 0.059 | 0.730 | -0.376 | 0.493 |
| believes people of my race are fairly treated in a health-care setting: Yes (vs. No) | 0.197* | 0.0851 | -0.112 | 0.506 |
| distrust in government | 0.361 | 0.110 | -0.198 | 0.920 |
| believes COVID-19 vaccine is tested for the safety of my race | 0.293 | 0.0933* | -0.135 | 0.720 |
| trust in vaccine development process of pharmaceutical firms | -0.111 | 0.603 | -0.705 | 0.482 |
| believes general vaccine is tested for the safety of my race | 0.437** | 0.0288 | -0.081 | 0.956 |
| believes in the importance of mask-wearing | 0.928*** | 0.000 | 0.467 | 1.389 |
| believes COVID is used by people for world manipulation | -0.310** | 0.0423 | -0.715 | 0.094 |
| **Observations R^2^**  **Adjusted R^2^**  **Residual Std. Error**  **F-Statistic** | 2454 0.793 0.785 1.730 (df=2362)  99.33*** (df=91; 2362) |  |  |  |

*Note:* *p<0.1; **p<0.05; ***p<0.01

**Table J3: The estimated regression coefficients of demographic factors**

| **variables** | **regression coefficients** | **p-value** | **lower 95% confidence interval** | **upper 95% confidence interval** |
| --- | --- | --- | --- | --- |
| covid status: had COVID (vs. never had COVID) | -0.110 | 0.460 | -0.402 | 0.182 |
| education: High school (vs. Bachelor's degree) | -0.068 | 0.650 | -0.362 | 0.226 |
| education: Less than high school (vs. Bachelor's degree) | 0.124 | 0.598 | -0.338 | 0.586 |
| education: Postgrad study/ professional degree (vs. Bachelor's degree) | -0.005 | 0.975 | -0.333 | 0.322 |
| education: Some college/associates (vs. Bachelor's degree) | -0.043 | 0.742 | -0.295 | 0.210 |
| is essential worker: Yes (vs. No) | -0.111 | 0.300 | -0.322 | 0.099 |
| gender: Woman (vs. Man) | 0.181* | 0.056 | -0.004 | 0.366 |
| income: $30,000 to $60,000 (vs. $100,000 or more) | -0.196 | 0.165 | -0.472 | 0.080 |
| income: $60,000 to under $100,000 (vs. $100,000 or more) | -0.216* | 0.077 | -0.455 | 0.023 |
| income: Less than $30,000 (vs. $100,000 or more) | -0.244* | 0.091 | -0.526 | 0.039 |
| political affiliation: Democrat (vs. Republican) | -0.006 | 0.970 | -0.302 | 0.291 |
| political affiliation: Independent (vs. Republican) | -0.194 | 0.147 | -0.457 | 0.068 |
| political affiliation: Other (vs. Republican) | -0.164 | 0.475 | -0.615 | 0.287 |
| census regions: Midwest (vs. West) | 0.094 | 0.475 | -0.163 | 0.351 |
| census regions: Northeast (vs. West) | 0.029 | 0.833 | -0.243 | 0.301 |
| census regions: South (vs. West) | 0.041 | 0.733 | -0.196 | 0.278 |
| generation: Gen X (vs. Boomer) | -0.190 | 0.171 | -0.462 | 0.082 |
| generation: Gen Z (vs. Boomer) | -0.199 | 0.366 | -0.630 | 0.232 |
| generation: Millennial (vs. Boomer) | -0.286** | 0.045 | -0.565 | -0.007 |
| generation: Silent (vs. Boomer) | -0.008 | 0.963 | -0.338 | 0.322 |
| comorbidity: at least one comorbidity (vs. no comorbidities) | -0.119 | 0.273 | -0.332 | 0.094 |
| race: 2+, non-Hispanic (vs. White) | 0.107 | 0.621 | -0.318 | 0.533 |
| race: Asian (vs. White) | 0.498** | 0.032 | 0.043 | 0.953 |
| race: Black (vs. White) | -0.043 | 0.833 | -0.442 | 0.357 |
| race: Hispanic (vs. White) | 0.020 | 0.890 | -0.257 | 0.296 |
| race: Other, non-Hispanic (vs. White) | -0.648 | 0.166 | -1.564 | 0.269 |
| urbanicity: Rural (vs. Urban) | -0.222 | 0.117 | -0.499 | 0.055 |
| **Observations R^2^**  **Adjusted R^2^**  **Residual Std. Error**  **F-Statistic** | 2454 0.793 0.785 1.730 (df=2362)  99.33*** (df=91; 2362) |  |  |  |

*Note:* *p<0.1; **p<0.05; ***p<0.01

**Table J4: The estimated regression coefficients of emotions, knowledge, social influences and constant**

| **variables** | **regression coefficients** | **p-value** | **lower 95% confidence interval** | **upper 95% confidence interval** |
| --- | --- | --- | --- | --- |
| **Emotions** |  |  |  |  |
| regret if I did not take COVID vaccine | 1.819*** | 0.000 | 1.360 | 2.279 |
| worries about COVID | 0.185 | 0.393 | -0.240 | 0.611 |
| felt depressed in the past 5 days | -0.122 | 0.513 | -0.488 | 0.244 |
| **Knowledge** |  |  |  |  |
| COVID-19 knowledge | 0.151 | 0.419 | -0.216 | 0.519 |
| general vaccine knowledge | -0.002 | 0.995 | -0.589 | 0.585 |
| **Social influence** |  |  |  |  |
| reference community**** thinks that threat of COVID-19 is exaggerated: I really have no idea (vs. No) | -0.165 | 0.317 | -0.487 | 0.158 |
| reference community thinks that threat of COVID-19 is exaggerated: Yes (vs. No) | 0.187 | 0.154 | -0.070 | 0.445 |
| reference community considers COVID-19 a serious threat: Not sure (vs. No) | 0.340 | 0.142 | -0.114 | 0.794 |
| reference community considers COVID-19 a serious threat: Yes (vs. No) | 0.164 | 0.317 | -0.157 | 0.484 |
| reference community expects me to take COVID-19 vaccine: Not sure or no answer (vs. No) | 0.572*** | 0.000 | 0.267 | 0.878 |
| reference community expects me to take COVID-19 vaccine: Yes (vs. No) | 0.362*** | 0.014 | 0.074 | 0.650 |
| proportion of community that I think will take the COVID-19 vaccine | 0.852*** | 0.000 | 0.401 | 1.303 |
| **Constant** | | | | |
| constant | 2.747*** | 0.000 | 1.781 | 3.713 |
| **Observations R^2^**  **Adjusted R^2^**  **Residual Std. Error**  **F-Statistic** | 2454 0.793  0.785 1.730 (df=2362)  99.33*** (df=91; 2362) |  |  |  |

*Note:* *p<0.1; **p<0.05; ***p<0.01

**** respondents mostly specified their immediate family and friends as their reference community

**Table J5: The estimated regression coefficients of influencers, outcome expectations, and structural factors**

| **variables** | **regression coefficients** | **p-value** | **lower 95% confidence interval** | **upper 95% confidence interval** |
| --- | --- | --- | --- | --- |
| **Influencers** |  |  |  |  |
| gets information on COVID-19 from church: Yes (vs. No) | 0.233 | 0.205 | -0.128 | 0.594 |
| gets information on COVID-19 from Fox News: Yes (vs. No) | -0.117 | 0.275 | -0.325 | 0.092 |
| gets information on COVID-19 from left-wing media: Yes (vs. No) | -0.013 | 0.902 | -0.216 | 0.191 |
| gets information on COVID-19 from scientists: Yes (vs. No) | 0.010 | 0.918 | -0.190 | 0.211 |
| gets information on COVID-19 from social media: Yes (vs. No) | -0.225* | 0.066 | -0.464 | 0.014 |
| believes in COVID-19 information from my preferred news channel | -0.234 | 0.317 | -0.693 | 0.225 |
| believes in COVID-19 information from my physician | 0.086 | 0.745 | -0.432 | 0.604 |
| believes in COVID-19 information from Trump | 0.088 | 0.660 | -0.303 | 0.478 |
| believes in COVID-19 information from my social media contacts | 0.195 | 0.424 | -0.283 | 0.674 |
| believes in COVID-19 information from my local community | 0.136 | 0.592 | -0.361 | 0.633 |
| believes in COVID-19 information from public health organizations | 0.677* | 0.096 | -0.120 | 1.475 |
| believes in COVID-19 information from public health officials | -0.584 | 0.153 | -1.385 | 0.218 |
| **Outcome expectations** |  |  |  |  |
| expected chance of getting long-term side-effects from COVID vaccine | -1.127*** | 0.003 | -1.864 | -0.390 |
| expected chance of getting short-term side-effects from COVID vaccine | -0.016 | 0.963 | -0.682 | 0.651 |
| expected chance of getting COVID with no vaccine | 0.680** | 0.043 | 0.022 | 1.337 |
| expected chance of getting seriously ill from COVID-19 | 0.272 | 0.550 | -0.620 | 1.165 |
| expected chance of dying from COVID with no vaccine | -0.175 | 0.586 | -0.803 | 0.454 |
| amount of control over getting COVID | -0.079 | 0.661 | -0.432 | 0.274 |
| **Structural enablers/barriers** |  |  |  |  |
| delayed medical care due to childcare: Yes (vs. No) | -0.117 | 0.684 | -0.682 | 0.447 |
| delayed medical care due to cost: Yes (vs. No) | 0.023 | 0.849 | -0.219 | 0.266 |
| delayed medical care due to transportation: Yes (vs. No) | -0.045 | 0.823 | -0.442 | 0.352 |
| delayed medical care due to work schedule: Yes (vs. No) | 0.103 | 0.414 | -0.144 | 0.349 |
| have personal care physician: Yes (vs. No) | -0.268* | 0.073 | -0.561 | 0.025 |
| easy to get COVID-19 vaccine once it is available: Yes (vs. No) | 0.055 | 0.549 | -0.125 | 0.234 |
| has health insurance: Yes (vs. No) | 0.269 | 0.116 | -0.066 | 0.604 |
| **Observations**  **R^2^**  **Adjusted R^2^**  **Residual Std. Error**  **F-Statistic** | 2454  0.793 0.785 1.730 (df=2362)  99.33*** (df=91; 2362) |  |  |  |

*Note:* *p<0.1; **p<0.05; ***p<0.01
